# Intersectional inequalities in healthcare utilisation in informal settlements in Freetown, Sierra Leone: a multilevel analysis of individual heterogeneity and discriminatory accuracy (MAIHDA)

**DOI:** 10.1186/s12939-026-02851-w

**Published:** 2026-04-14

**Authors:** Ibrahim Juldeh Sesay, Samira Sesay, Haja Wurie-Kamara, Sia Morenike Tengbe, Sullaiman Fullah, Dora Vangahun, Ibrahim Gandi, Noemia Teixeira de Siqueira Filha, Rajith Lakshman, Abu Conteh, Samuel Saidu, Braima Koroma, Bintu Mansaray, Helen Elsey, Lana Whittaker, Laura Dean, Neele Wiltgen Georgi, Motto Nganda, Francis Refell, Joseph M. Macarthy, Alastair H. Leyland, Rachel Tolhurst, Eliud Kibuchi

**Affiliations:** 1 Sierra Leone Urban Research Center, Freetown, Sierra Leone; 2 College of Medicine and Allied Health Sciences, University of Sierra Leone, Freetown, Sierra Leone; 3 Centre of Dialogue on Human Settlement and Poverty Alleviation, Freetown, Sierra Leone; 4 Department of Health Sciences, Hull and York Medical School, University of York, York, UK; 5 Institute of Development Studies (IDS), Falmer, UK; 6 Department of International Public Health, Liverpool School of Tropical Medicine, Liverpool, UK; 7 School of Health and Wellbeing, University of Glasgow, Clarice Pears Building, 90 Byres Road, Glasgow, G12 8TB UK

**Keywords:** Healthcare utilisation, Quantitative intersectionality, Informal settlements, MAIHDA, Intersectional inequalities

## Abstract

**Introduction:**

Residents of informal settlements face significant intersectional inequalities, due to the overlapping and compounding effects of multiple social factors. This study aims to explore how these intersecting social factors, identified by community members, combine to shape household-level inequalities healthcare utilisation (HU) among residents of informal settlements in Freetown, Sierra Leone.

**Methods:**

This study employed participatory action research to collaboratively identify key social determinants affecting healthcare utilisation in Freetown’s informal settlements. A cross-sectional health and wellbeing survey was implemented in April-May 2023 and collected data from 4,871 households in Cockle Bay, Dwazark, and Moyiba informal settlements. The survey questions were codesigned by researchers and community fieldworkers, informed by prior qualitative research. Two outcomes were analysed: HU within the settlement (*n* = 4,821), and outside the settlement (*n* = 4,616). A multilevel analysis of individual heterogeneity and discriminatory accuracy (MAIHDA) was conducted, nesting households within 122 intersectional strata. These strata were defined by six social factors: head of household gender, marital composition, engagement in income-generating activity, food security, disability and the household’s settlement. Intersectional measures included variance partition coefficient (VPC), the proportional change in variance (PCV), and residual intersectional effects.

**Results:**

VPCs of 0.9% (PCV, of 92.8%) for HU within the settlements and of 3.9% (PCV, 81.7%) for HU outside the informal settlements suggest moderate but meaningful intersectional effects in shaping HU inequalities. The lowest levels of HU within informal settlements were observed among single, male, disabled individuals in Moyiba who lacked income-generating activities and experienced food insecurity. For HU outside the settlement, the lowest levels were found among female-headed households in Moyiba who were married, cohabiting, or engaged with a disabled household member, experienced food insecurity, and were engaged in income-generating activities.

**Conclusion:**

This study identifies and quantifies inequalities in HU at the household level across three informal settlements in Freetown, driven by intersecting social factors. Addressing these inequalities requires policies that are universally accessible but implemented with an intensity proportionate to the level of vulnerability, ensuring that support is targeted to those most in need.

**Supplementary Information:**

The online version contains supplementary material available at 10.1186/s12939-026-02851-w.

## Highlights

The findings identify and quantify intersectional inequalities in healthcare utilisation (HU), particularly among the most vulnerable groups.

Single women without income and single men with disabilities and no income in Moyiba were less likely to utilise healthcare.

Households with protective factors against illness showed higher HU than those exposed to illness-enabling conditions.

Stakeholders are encouraged to address HU inequalities through social security and health insurance, proportionately targeted by level of need.

**Supplementary Information:**

The online version contains supplementary material available at 10.1186/s12939-026-02851-w.

## Background

The global urban population is projected to exceed 60% by 2030, with the most significant increase expected in lower and middle income (LMICs) countries [47, 52]. Over a billion informal urban dwellers live in informal settlements, commonly known as slums [52]. These informal urban dwellers often face poverty, inadequate housing, and limited social protection [44] which exposes them to heightened health, social, and financial vulnerabilities compared to other urban and rural populations [18, 39, 45].

Healthcare utilisation (HU), defined as accessing health service(s) when needed [3, 49] is particularly challenging for informal urban dwellers due to extreme poverty, limited health literacy, and social exclusion [2, 31, 38]. Typically, HU is influenced by sociodemographic characteristics (e.g., age, sex, marital status), enabling factors which are defined as resources and conditions that facilitate or hinder access (e.g., health system and policy, cultural, religion, financing, organisation); and need factors, which refer to perceived or evaluated health status that drives individuals to seek healthcare (e.g., illness, personal awareness, attitude and perception) [3, 28, 30, 38, 49].

Informal settlements often lack public health facilities, and those available are few, under-resourced, and have inconvenient hours with majority of urban dwellers, particularly the poorest relying on pharmacies and drug sellers [14, 50]. A scoping review identified key factors influencing HU in informal settlements as residency time, financial constraints, social support, physical environment, nature of health systems, sociocultural expectations, and stigma [38].

In Freetown, Sierra Leone, 60% of the one million residents live in 68 informal settlements, occupying only 36% of the residential area [19, 20]. Despite this, HU inequalities among these populations are poorly understood [25]. Research from other countries shows that informal dwellers, often among the most vulnerable urban populations, usually have poor access to health services with reported high use of pharmacies and drug sellers [2, 38, 50].

A report in three informal settlements (Cockle Bay, Dwazark and Moyiba) found that HU in public and private healthcare providers was 40%, followed by drug(i.e., medicine) peddlers and private nurses at 40% [41]. However, healthcare providers visited by respondents varied across three informal based on whether health facilities were available [41]. For example, in Cockle Bay 60% of respondents used private healthcare and 32% relied on drug(i.e., medicine) sellers due to the absence of public healthcare. However, in Moyiba 74% of respondents used public healthcare, while only 18% and 12% relied on drug sellers and private services respectively [41].

A study in two of Freetown’s informal settlements found that factors like gender, marital status, household composition, poverty, and tenure housing affected healthcare access [9]. In 2020, a qualitative, exploratory phase of a Community Based Participatory Research (CBPR) study in three informal settlements (Cockle Bay, Dwazark, and Moyiba) identified HU as a key issue, influenced by disability, presence of chronic health condition, food security, gender, and age [40]. However, no studies have examined how the complex interplay of these multiple factors combine to produce intersectional inequalities in HU in informal settlements.

Intersectional analysis helps to understand how combinations of social factors contribute to intersectional inequalities [10, 11, 16]. Intersectionality theory posits that social positions, formed through the interaction of multiple identity dimensions, are interdependent and mutually constitutive, illustrating how interconnected systems of power and identity produce inequalities [10, 11]. Generally, intersectional inequalities refer to disparities that arise from the overlapping and interacting effects of multiple social dimensions, compounded by structural barriers [10, 11].

The use of quantitative intersectionality in social epidemiology has grown, particularly through multilevel analysis of individual heterogeneity and discriminatory accuracy (MAIHDA) [34]. MAIHDA treats social positions multiplicatively, not additively, highlighting how intersecting power dynamics create specific health vulnerabilities [34]. We employed the MAIHDA approach to investigate intersectional inequalities in HU at the household level, using data from the 2023 Freetown Informal Settlements Health and Wellbeing Survey [41]. Our aim was to analyse HU inequalities based on household-level social factors identified by community members during the qualitative exploratory study: head of household gender, household’s marital composition, household engagement in an income generating activity, household’s food security, disability in household, and informal settlement [40].

Importantly, while HU is conceptually defined as accessing healthcare when needed [49], our analyses focused on actual healthcare accessibility. That is, ability of households to obtain healthcare when required, rather than perceived need or intent [29]. By adopting a broader framework of healthcare accessibility, we concentrate on the demand side of the healthcare system, which is influenced by factors such as disease burden, health-related knowledge and attitudes, and self-care practices [29]. This approach enables examination of differences in healthcare-seeking inequalities across intersectional household profiles. However, we acknowledge that healthcare accessibility data alone does not account for unmet need and may confound health needs with access.

To ensure the robustness of household-level factors considered in creating social strata, we cross-referenced them with existing literature. Female-headed households are three times less likely to seek healthcare compared to male-headed in Nairobi informal settlements [37]. In Kerman slums, Iran, married individuals were more likely to seek healthcare than single people [2]. People with disabilities in informal settlements tend to use healthcare services more than those without disabilities [38]. Residents involved in income-generating activities in informal settlements are more likely to seek healthcare compared to those who are not [38]. The specific informal settlement where a household resides also influences HU through social and cultural factors such as social support and nature of healthcare systems [35]. Households facing food insecurity report the highest unmet healthcare needs compared to those without food insecurity in 38 Indian slums [24]. This intersectional analysis aims to inform HU policy interventions in Freetown’s informal settlements.

## Methodology

### Study setting

This study was conducted in three informal settlements in Freetown, Sierra Leone: Cockle Bay, Moyiba, and Dwazark. Sierra Leone, located in West Africa, has one of the lowest Human Development Index (HDI) scores, highlighting significant challenges in health, education, and living standards [27, 51]. These settlements were purposively selected for their diverse spatial, social, and economic characteristics, and the findings are therefore not generalisable beyond the study areas.

Moyiba and Dwazark are hillside settlements in the eastern and central parts of Freetown, respectively, both facing perennial challenges of healthcare access [27]. Dwazark, with an estimated 16,500 residents in 2018, has nearly 65% of its population under 30 years old [27]. Land ownership disputes in Dwazark have led to poor housing, inadequate roads, and sanitation issues due to limited infrastructure development [27]. Most residents are involved in informal employment, such as petty trading and stone mining. Most Dwazark residents used public (88%) and private (77%) healthcare facilities, followed by drug sellers (54%) and private nurse (52%) [41].

Moyiba, home to around 37,000 residents, is marked by low income levels, poor environmental conditions, housing challenges, and high unemployment, with many engaged in stone quarrying and petty trading [27]. The majority of Moyiba residents sought healthcare from public health facilities (82%), drug sellers (62%), private nurses (45%), and traditional healers (37%) [41].

Cockle Bay, located along the Aberdeen Creek on the western coast, is a low lying settlement with approximately 16,000 residents [1, 27]. The land is owned by the local municipality, and the settlement suffers from poor drainage and infrastructure, making it highly vulnerable to flooding, waterborne diseases (especially cholera), and fires. Most residents rely on fishing and cockle production for their livelihoods [27]. Cockle Bay residents relied on drug sellers (56%) and private facilities (35%) to access healthcare, with 55% not seeking care due to lack of available health facilities [41].

### Data

We used data from the 2023 Freetown Informal Settlements Health and Wellbeing Survey, conducted in Cockle Bay, Dwazark, and Moyiba between April to May 2023, targeting adults 18 years and older [41]. The survey questions were co-designed by researchers and community-based fieldworkers, informed by findings from a prior participatory and qualitative exploratory study in 2020 and 2021 as part of The GCRF Accountability for Informal Urban Equity Hub (ARISE) [4, 41]. Residents identified HU as a key issue, highlighting six factors influencing HU at the household level: head of household gender, marital composition, engagement in income-generating activities, food security, disability, and the specific informal settlement [40].

The sample size was determined based on the estimated proportion of households facing barriers to HU, as measured by the question “Do you or your household members face any barriers to accessing healthcare?” This proportion was 0.47, based on a pilot survey involving 159 respondents. We used a margin of error of 0.03, and a design effect of 10 to account for the hybrid sampling technique (combining probability and non-probability sampling due to lack of effective sampling frame), a critical value of 0.05 (95% confidence interval), and a nonresponse rate of 10%. The final sample size of 4,883 households using Eq. (1) from [7].

1 $$\:n=\frac{{z}_{1-\frac{\alpha\:}{2}}^{2}\times\:p\times\:\left(1-p\right)\times\:deff}{{d}^{2}\times\:\left(1-NR\right)}$$

Where: *n* = required sample size; *p* = proportion of households facing barriers to HU (0.47); *deff* = design effect(10); *d*= margin of error (0.03); $\:{z}_{1-\frac{\alpha\:}{2}}^{2}\:$= critical value for the standard normal distribution corresponding to a Type 1 error rate of a two-tailed test (1.96); *and NR* = non-response rate (10%). Although the sample size was calculated based on the estimated proportion of households facing barriers to HU, it provided sufficient statistical power to examine intersectional inequalities in HU for the current analysis, as this research question required a smaller sample size.

The selection of households for interviews was conducted in multiple stages. First, households were proportionally allocated based on 2018 estimated population: Cockle Bay (*n* = 1,251), Dwazark (*n* = 1,321), and Moyiba (*n* = 2,312). Each settlement’s households were then equally distributed across different zones: Cockle Bay (313 households per zone x 4 zones), Moyiba (232 households per zone x 10 zones), and Dwazark (111 households per zone x 12 zones). In each zone, a random household along the chosen outward direction from an identified landmark was selected as the starting point for interviews. Landmarks used included mosques, cinemas, community centres and water points with settlement boundaries identified during a GIS mapping study conducted earlier [40].

Next, the closest household to the landmark was selected, and interviews were conducted with every $\:{k}^{th}$ household until the zone boundary was reached. The $\:{k}^{th}$ value was calculated by dividing the number of households by the number of landmarks in that zone. One consenting adult (18 years and older), either the head of the household or the most senior household member was interviewed in each household.

The interviews were conducted face-to-face by 35 co-researchers and 18-community mobilisers, all recruited from the study areas for their familiarity with the communities. The survey team underwent a 4-day training program covering survey procedures, questionnaire content, the REDCap tool, ethical considerations in research, safeguarding, and sampling techniques. A total of 5,121 households were initially interviewed. After data cleaning with co-researchers, which removed 250 (4.9%) duplicate records, the final sample comprised 4,871 complete records.

Given the hybrid sampling design and the absence of a comprehensive sampling frame, inference is limited to the three informal settlements. Generalizability to other informal settlements should be approached with caution.

### Outcome

We considered two primary outcomes. The first was HU within the settlement, assessed whether the respondent or any household member sought healthcare services (both formal and informal) within their informal settlement in the past month. This was measured using the question “Did you or any member of your household seek health provision service during the last one month within your community?“ This measure helps identify groups likely to face inequalities when accessing locally available healthcare services, which are often characterised by constrained resources and a lack of formal health facilities. Formal healthcare providers are licensed, regulated professionals and institutions which offer healthcare services [36, 41]. These include public hospitals, health clinics, private clinics, and pharmacies. Informal healthcare providers, on the other hand, are typically unlicensed individuals or those with limited formal medical training who deliver care outside the formal health system [36]. They include traditional healers, medicine peddlers, and private nurses.

The second outcome, HU outside the informal settlement, examined whether respondent or any household member sought healthcare services (both formal and informal) outside their informal settlements during the past month. This was measured using the question “Did you or any member of your household seek health provision service during the last one month outside your community?“ Individuals often seek healthcare outside their informal settlements due to limited infrastructure, poor quality of care, concerns about privacy and stigma, and accessibility challenges [38]. This outcome highlights the vulnerabilities and inequalities households face when accessing healthcare beyond their immediate environment.

Considering that data were collected in April and May, the period during which individuals and their household members sought healthcare falls within March, April, and early May. This time frame coincides with late dry season, which is characterised by reduced rainfall, more reliable transportation and improved accessibility to health facilities. However, it is also associated with increased dust-related illnesses and heighted water scarcity, both of which contribute to elevated health risks.

A sensitivity analysis was conducted by combining HU within and outside informal settlements to provide a more comprehensive view of HU inequalities households face when accessing healthcare services both locally and beyond. Additionally, we conducted a separate sensitivity analysis focused solely on HU involving formal providers, to identify any differences between overall HU and utilisation specific to formal providers.

### Social position dimensions

The variables used to create household-level intersectional strata were informed by the community members and also cross-referenced them with literature to ensure their validity. These variables are head of household gender (*female*,* male*), marital composition (*single*,* married/cohabitating/engaged*,* divorced/separated/widowed*), engagement in income-generating activity *(yes*,* no*), food security (*food secure*,* food insecure*), disability (*yes*,* no*), and household’s settlement (*Cockle Bay*,* Dwazark*,* Moyiba*). Households were considered food secure if they were able to eat the kinds of the food they preferred or if they rarely (once or twice) failed to eat due to lack of resources in the past one month. Food insecure households were those that reported sometimes (3 to 10 times) or often (more than 10 times) failing to eat their preferred food due to a lack of resources.

### Explanatory variables

In addition to adjusting for factors influencing HU as identified by community members, we also controlled for other household-level variables that are associated with HU based on literature [38]. This approach aims to better understand HU inequalities by ensuring that observed intersectional differences are not obscured or exaggerated by other household-level sources of inequality.

The explanatory variables considered include factors that predispose households to utilise healthcare services such as household size, length of residence, household tenure, water sources (i.e., *piped dwelling*,* piped neighbor*,* piped compound*,* public tap/standpipe*,* compound well*,* spring water*,* rainwater*,* bowser water*,* kiosk water*,* bottled water*,* sachet water*,* surface water*,* neighbor’s well*,* other water sources*), water distance, water shortage, types of toilet (i.e., *flush*,* pit latrine*,* bucket*,* hanging*,* flying*,* open defecation*,* other*), shared toilet, toilet access, waste disposal (i.e., *community*,* around house*,* dumping site*,* drainage*,* solid waste collectors*,* sea*,* Other areas*), waste payment, environmental disaster).

Moreover, we considered enabling factors that may hinder or facilitate HU, such as sources of household income (i.e., *business*,* fishing*,* government salaried*,* private salaried*,* informal salaried*,* daily wage*,* bike ride*,* stone mining*,* other sources*).

The full description of variables used in the analyses is in Supplementary Table 1a.

### Statistical methods

Descriptive statistics for categorical variables were reported as frequencies and percentages. We used the MAIHDA approach to estimate intersectional inequalities in HU, clustering households within social strata [15, 16, 17, 34]. In the MAIHDA, households (level 1) are nested within social strata (level 2). Traditional fixed effects models for quantitative intersectionality have limitations: they assume independent, not joint, effects of social positions; limit interaction terms due to sample size; and require a reference group, constraining evaluations of inequalities [34].

Using the MAIHDA framework, we assessed HU inequalities through three multilevel logistic models Model 1 (null model) included only an intercept and the random effect of social strata, without any main effects [26, 34]. The variance partition coefficient (VPC) estimated in Model 1 represented the proportion of the total variance attributable to differences across social strata [22, 34]. A VPC above 5% indicates an acceptable discriminatory accuracy of significant differences in HU between intersectional strata, and similar HU within strata [22].

Model 2 (main effects) included the social strata variables as additive main effects to assess how much of the intersectional effect is explained additively [34]. The VPC for Model 2 captures the unexplained variance remaining in the intersectional differences after accounting for these main effects. A VPC of 0% would indicate that all intersectional inequalities in HU are fully explained by the main effects. Since our models are logistic, we used a latent approach to compute VPC by assuming a residual variance of $\:{\pi\:}^{2}/3=3.29\:$ [26]. Several studies have shown that a VPC that between 5% and 20%, depending on the outcome and population, reflects significant and meaningful intersectional effects [17]. We also calculated the proportional change in variance (PCV) between Model 1 and Model 2 to quantify the percentage of intersectional variance explained after including additive main effects [15, 34]. A lower PCV suggests that more intersectional variance remains unexplained and is attributable to intersectional effects. PCV for Model 2 is derived from the difference in VPCs between Models 1 and 2.

Model 3 (main effects and explanatory variables) included both social strata variables as main effects and additional explanatory variables. This model helps assess the independent effects of social disadvantage by examining how much variation in HU is explained by social strata, while controlling for other relevant household-level factors. In other words, if HU remains significantly associated with social strata in Model 3, it suggests that intersectional inequalities in HU persist beyond what is explained by the included explanatory variables. The VPC for Model 3 reflects the unexplained variance remaining in intersectional differences after accounting for both social strata and explanatory variables. The PCV for Model 3 was derived from the VPCs of Models 1 and 3, capturing the reduction in intersectional variance due to the inclusion of household-level social strata and explanatory variables.

VPC and PCV were used to assess the changes in the variance explained by inclusion of variables across models. Coefficients and 95% confidence interval (95%CI) were presented as odds ratios (OR). Predicted intersectional residual effects were plotted to examine whether inequalities are intersectional and extend beyond additive effects. Positive residuals indicate higher HU than expected, while negative residuals indicate lower HU than expected. Residuals with confidence intervals that do not contain zero suggest a significant deviation from the additive expectation. This implies that the observed HU for a given stratum (either positive or negative) cannot be fully explained by the individual social factors alone, but rather by their intersectional combinations [17]. No weights and finite population corrections were applied in the analysis.

We used Stan for full Bayesian inference through Markov Chain Monte Carlo (MCMC) methods to fit MAIHDA models [6]. The models were fitted using ‘brms ‘package for Bayesian multilevel models using Stan (version 2.16.1) [5] in R statistical software (version 4.0.12) [46]. To avoid model overfitting and stabilise estimates, we applied regularising priors for both fixed and random effects [32, 33]. For fixed effects, we used a normal prior with mean 0 and standard deviation 1 $\:(i.e.,\:N\left(\mathrm{0,1}\right)$, which encourages shrinkage of estimates towards zero [32]. For the random intercept, we applied a student’s t-distribution with 2 degrees of freedom, centered at 0, and scaled by 10 (i.e., $\:Studen{t}^{{\prime\:}}s\:t(v=1,\mu\:=0,\:\sigma\:=10)$. This distribution allows for more extreme values while still promoting shrinkage towards the center, providing a balance between flexibility and stability [32, 33].

We ran 20,000 iterations with a burn-in period of 2,000. Posterior diagnostics were assessed using the Rhat statistic, where values less than 1.01 indicate good convergence [33]. We also evaluated sampling efficiency [33]. This was done by examining the center of posterior distribution using the Bulk Effective Sample Size (ESS), with values greater than or equal to 1000 indicating reliable estimates. To assess sampling efficiency in the tails of the posterior distribution, we used Tail ESS, where values above 1000 suggest reliable estimation of extreme quantile [33]. MCMC chains were checked graphically for convergence. The R codes are available at https://github.com/Kibuchi-eliud/Inequalities-in-healthcare-utilisation-in-informal-settlements-in-Freetown-Sierra-Leone-.git in GitHub.

### Ethical considerations

The ethical approval for the data collection was obtained from the Sierra Leone Ethics and Scientific Review Committee (SLESRC: 026/04/2023) and the Liverpool School of Tropical Medicine (21 − 007).

## Results

Table 1 summarises the study sample characteristics for HU within the settlement and HU outside the settlement. Overall, 29.8% of households utilised healthcare within the settlement and 32.1% outside the settlement in the past month during the interview period. HU within the settlement was slightly higher for female headed households (31.1%) compared to male headed (29.3%). However, male headed households had slightly higher HU outside the settlement (33.1%) compared to female headed households (29.5%). HU within the settlement for households with disabled persons was higher (37.4%) compared to those without (29.3%). Similarly, HU outside the settlement for households with disabled persons was higher (42.6%) compared to those without (31.4%).

HU within the settlement was highest for households with a divorced/separated/widowed head (32.4%), followed by those with a married/cohabiting/engaged head (30.6%) and single person households (26.5%). Differences in HU outside the settlement were smaller, with households headed by married/cohabiting/engaged persons having the highest HU (32.9%) followed by single person households (31.9%) and those headed by divorced/separated/widowed individuals (30.1%). HU within the settlement was higher for households involved in income-generating activities (30.9%) compared to those without (26.5%). HU outside the settlement was also higher for households engaged in income-generating activities (32.8%) compared to those without (29.8%).

HU within the settlement was higher for food insecure households (33.9%) compared to food secure households (25.7%). Food insecure households also utilised healthcare outside the settlement more frequently (39.2%) compared to food-secure households (24.9%). Amongst the settlements, HU within the settlement was highest in Dwazark (46.4%), followed by Cockle Bay (25.3%) and Moyiba (22.2%). In contrast, HU outside the settlement was highest in Cockle Bay (57.1%), more than double of Dwazark (25.9%) and Moyiba (23.9%).

**Table 1:** Household characteristics by healthcare utilisation (HU) within and outside informal settlements in Cockle Bay, Dwazark, and Moyiba

Variable name		Category	HU within settlement: Frequency (%)			HU outside settlement: Frequency (%)		
			No	Yes	Total	No	Yes	Total
Head of household gender		Female	914(68.9)	412(31.1)	1326(27.5)	938(70.5)	392(29.5)	1330(27.5)
		Male	2470(70.7)	1025(29.3)	3495(72.5)	2347(66.9)	1161(33.1)	3508(72.5)
Disability in household		No	3185(70.7)	1318(29.3)	4503(93.4)	3102(68.6)	1417(31.4)	4519(93.4)
		Yes	199(62.6)	119(37.4)	318(6.6)	183(57.4)	136(42.6)	319(6.6)
Family type		Single	892(73.5)	322(26.5)	1214(25.2)	380(68.1)	178(31.9)	558(11.5)
		Married/cohabit/ engaged	2115(69.4)	934(30.6)	3049(63.2)	2050(67.1)	1007(32.9)	3057(63.2)
		Divorced/separated/widowed	377(67.6)	181(32.4)	558(11.6)	855(69.9)	368(30.1)	1223(25.3)
Income activity engagement		No	870(73.5)	314(26.5)	1184(24.6)	835(70.2)	355(29.8)	1190(24.6)
		Yes	2514(69.1)	1123(30.9)	3637(75.4)	2450(67.2)	1198(32.8)	3648(75.4)
Food security		Food secure	1779(74.3)	615(25.7)	2394(49.7)	1805(75.1)	599(24.9)	2404(49.7)
		Food insecure	1605(66.1)	822(33.9)	2427(50.3)	1480(60.8)	954(39.2)	2434(50.3)
Community residence		Cockle Bay	830(74.8)	280(25.3)	1110(23.0)	478(42.9)	636(57.1)	1114(23.0)
		Dwazark	748(53.9)	641(46.2)	1389(28.8)	1030(74.1)	360(25.9)	1390(28.7)
		Moyiba	1806(44.8)	516(22.2)	2322(48.2)	1777(76.1)	557(23.9)	2334(48.2)
Length of residence		0–1 years	26(74.4)	92(25.6)	359(7.5)	246(68.3)	114(31.7)	360(7.4)
		1–5 years	1073(74.9)	360(25.1)	1433(29.7)	978(67.9)	463(32.1)	1441(29.8)
		6–10 years	696(72.3)	267(27.7)	963(19.9)	565(67.8)	311(32.2)	967(19.9)
		More than 10 years	1348(65.3)	718(34.8)	2066(42.8)	1405(67.9)	665(32.2)	2070(42.8)
Household tenure		Tenant	1907(68.6)	872(31.4)	2779(57.6)	1890(37.8)	899(32.2)	2789(57.7)
		landlord	1041(72.7)	391(27.3)	1432(29.7)	966(67.3)	470(32.7)	1436(29.7)
		Free living	342(71.3)	138(28.8)	480(9.9)	343(71.2)	139(28.8)	482(10.0)
		Caretaker/lease/temporary stay/others	94(72.3)	36(27.7)	130(2.7)	96(65.7)	45(34.4)	131(2.7)
Water Sources	Piped dwelling	No	3261(69.9)	140.(30.1)	4664(96.7)	3181(68.0)	1498(32.0)	4679(96.7)
		Yes	123(78.3)	34(21.7)	157(3.3)	104(65.4)	55(34.6)	159(3.3)
	Piped neighbor	No	3186(71.2)	1289(28.8)	4475(92.8)	3128(69.7)	1361(30.3)	4489(92.8)
		Yes	198(57.2)	148(42.8)	346(7.2)	157(45.0)	192(55.0)	349(7.2)
	piped compound	No	3294(70.4)	1384(29.6)	4678(97.0)	3199(68.2)	1495(31.9)	4694(97.0)
		Yes	90(62.9)	53(37.1)	143(2.97)	86(59.7)	58(10.3)	144(2.9)
	Public tap/standpipe	No	2571(68.5)	1180(31.5)	3751(77.8)	2553(67.8)	1212(32.2)	3765(77.8)
		Yes	813(76.0)	257(24.0)	1070(22.2)	732(68.2)	341(31.8)	1073(22.2)
	Compound well	No	2943(71.9)	1152(28.1)	4095(84.9)	2777(67.6)	1332(32.4)	4109(84.9)
		Yes	441(60.7)	285(39.3)	726(15.1)	508(69.7)	221(30.3)	729(15.1)
	Spring water	No	2674(70.1)	1139(29.9)	3813(79.1)	2639(69.0)	1188(31.0)	3827(79.1)
		Yes	710(70.4)	298(29.6)	1008(20.9)	646(63.9)	365(36.1)	1011(21.0)
	Rainwater	No	2181(69.8)	943(30.2)	3124(64.8)	2001(63.8)	1134(36.2)	3135(64.8)
		Yes	1203(70.9)	494(29.1)	1697(35.2)	1284(75.4)	419(24.6)	1703(35.2)
	Bowser water	No	334(70.3)	1408(29.8)	4732(98.2)	3211(67.6)	1538(32.4)	4749(98.2)
		Yes	60(67.4)	29(32.6)	89(1.8)	74(83.2)	15(16.9)	89(1.8)
	Kiosk water	No	3162(69.8)	1366(30.2)	4528(93.9)	3093(68.1)	150(31.9)	4543(94.0)
		Yes	222(75.8)	71(24.2)	293(6.1)	192(65.1)	103(34.9)	295(6.1)
	Bottled water	No	3328(70.0)	1427(30.0)	4755(98.6)	3249(68.1)	1523(31.9)	4772(98.6)
		Yes	56(84.8)	10(15.2)	66(1.4)	36(54.6)	30(45.5)	66(1.4)
	Sachet water	No	1327(65.9)	687(34.1)	2014(41.8)	1493(73.8)	530(26.2)	2023(41.8)
		Yes	2057(73.3)	750(26.7)	2807(58.2)	1792(36.7)	1023(36.3)	2815(58.2)
	Surface water	No	2966(69.2)	1322(30.8)	4288(88.9)	2840(66.1)	1459(33.9)	4299(88.9)
		Yes	418(78.4)	115(21.6)	533(11.1)	445(82.6)	94(17.4)	539(11.1)
	Neighbor’s well	No	2900(71.1)	1177(28.9)	4077(84.6)	2856(69.8)	1237(30.2)	4093(84.6)
		Yes	484(65.1)	260(35.0)	744(15.4)	429(57.6)	316(42.4)	745(15.4)
	Other water sources	No	3327(70.1)	1419(29.9)	4746(98.4)	3243(68.1)	1520(31.9)	4763(98.5)
		Yes	57(76.0)	18(24.0)	75(1.6)	42(56.0)	33(44.0)	75(1.6)
Water distance		Less 30 min	1022(66.8)	508(33.2)	1530(31.7)	932(60.7)	604(30.3)	1536(31.8)
		30 min–1 h	1044(71.4)	418(28.6)	1462(30.3)	1060(72.3)	407(27.7)	1467(30.3)
		1–2 h	478(73.2)	175(26.8)	653(13.5)	449(68.6)	206(31.5)	655(13.5)
		Over 2 h	781(73.7)	279(26.3)	1060(21.9)	753(70.8)	311(29.2)	1064(22.0)
		Don’t know	59(20.9)	57(49.1)	116(2.4)	91(78.4)	25(21.6)	116(2.4)
Water shortage		Yes	1991(67.7)	948(32.3)	2939(60.9)	2025(68.8)	918(31.2)	2943(60.8)
		No	1386(74.0)	487(26.0)	1873(38.8)	1251(66.4)	634(33.6)	1885(38.9)
		Don’t know	7(77.8)	2(22.2)	9(0.2)	9(90.0)	1(10.0)	10(0.2)
Types of toilets	Flush	No	2595(70.7)	1078(29.3)	3673(76.2)	2657(72.0)	1034(28.0)	3688(76.2)
		Yes	789(68.7)	359(31.3)	1148(23.8)	628(54.6)	522(45.4)	1150(23.8)
	Latrine	No	1141(71.9)	447(28.2)	1588(32.9)	870(54.6)	723(45.4)	1593(32.9)
		Yes	2243(69.4)	990(30.6)	3233(67.1)	2415(74.4)	830(25.6)	3245(67.1)
	Bucket	No	2186(66.5)	1101(33.5)	3287(68.2)	2160(65.6)	1134(34.4)	3294(68.1)
		Yes	1198(78.1)	336(21.9)	1534(31.8)	1125(72.9)	419(27.1)	1544(31.9)
	Hanging	No	3172(70.0)	1357(30.0)	4529(93.9)	3134(68.9)	1412(31.1)	4546(93.9)
		Yes	212(72.6)	80(27.4)	292(6.1)	151(51.7)	141(48.3)	292(6.0)
	Flying	No	3368(70.2)	1431(29.8)	4799(99.5)	3272(67.9)	1544(32.1)	4816(99.6)
		Yes	16(72.7)	6(27.3)	22(0.5)	13(59.1)	9(40.9)	22(0.5)
	Open defecation	No	3318(70.3)	1400(29.7)	4718(97.8)	3249(68.7)	1483(31.3)	4732(97.8)
		Yes	66(64.1)	37(35.9)	103(2.1)	36(34.0)	70(66.0)	106(2.2)
	Other	No	3361(10.2)	1430(29.9)	4791(99.4)	3267(68.0)	1541(32.0)	4808(99.4)
		Yes	23(46.7)	7(23.3)	30(0.6)	18(60.0)	12(40.0)	30(0.6)
Shared toilet		Yes	2313(68.1)	1085(31.9)	3398(70.5)	2372(69.5)	1041(30.5)	3413(70.5)
		No	1030(75.0)	344(25.0)	1374(28.5)	883(64.2)	492(35.8)	1375(28.4)
		Not applicable	41(83.7)	8(16.3)	49(1.0)	30(30.0)	20(40.0)	50(1.0)
Toilet access		Yes	2864(70.0)	1226(30.0)	4090(84.8)	2793(68.1)	1311(31.9)	4104(84.8)
		No	502(71.0)	205(29.0)	707(14.7)	470(84.4)	238(33.6)	708(14.6)
		Don’t know	18(75.0)	6(25.0)	24(0.5)	22(84.6)	4(15.4)	26(0.5)
Waste disposal areas	Community	Yes	121(68.4)	56(31.6)	177(3.7)	112(63.3)	65(36.7)	177(3.7)
		No	3242(70.2)	1376(29.8)	4618(95.7)	3150(68.0)	1484(32.0)	4634(95.8)
		Don’t know	21(80.8)	5(19.2)	26(0.5)	23(85.2)	4(14.8)	27(0.6)
	Around house	No	1640(70.8)	675(29.2)	2315(48.0)	1387(59.8)	934(40.2)	2321(47.9)
		Yes	1744(69.6)	762(30.4)	2506(51.9)	1898(75.4)	619(24.6)	2517(52.0)
	Dumping site	No	3158(70.4)	1329(29.6)	4487(93.1)	3044(67.6)	1458(32.4)	4502(93.1)
		Yes	226(67.7)	108(32.3)	334(6.9)	241(71.7)	95(28.3)	336(6.9)
	Drainage	No	2642(70.4)	1110(29.6)	3752(77.8)	2511(66.7)	1252(33.3)	3763(77.8)
		Yes	742(69.4)	327(30.6)	1069(22.2)	774(72.0)	301(28.0)	1075(22.2)
	Solid waste collectors	No	2731(69.5)	1199(30.5)	3930(81.5)	2612(66.2)	1332(33.8)	3944(81.5)
		Yes	653(73.3)	238(26.7)	891(18.5)	673(75.3)	221(24.7)	894(18.5)
	Waste sea	No	2554(69.1)	1143(30.9)	3697(76.7)	2777(74.9)	933(25.2)	3710(76.7)
		Yes	830(73.8)	294(26.2)	1124(23.3)	508(45.0)	620(55.0)	1128(23.3)
	Waste others	No	3299(70.9)	1352(29.1)	4651(96.5)	3176(68.0)	1492(32.0)	4668(96.5)
		Yes	85(50.0)	85(50.0)	170(3.5)	109(64.1)	61(35.9)	170(3.5)
Waste payment		No	2663(69.7)	1156(30.3)	3819(79.2)	2551(66.5)	1283(33.5)	3834(79.3)
		Yes	721(72.0)	281(28.0)	1002(20.8)	734(73.1)	270(26.9)	1004(20.7)
Environmental disaster		No	197(55.7)	157(44.4)	354(7.34)	239(67.1)	117(32.9)	356(7.4)
		Yes	3187(71.4)	1280(28.7)	4467(92.7)	3046(68.0)	1436(32.0)	4482(92.6)
Sources of household income	business	No	2147(69.8)	931(30.2)	3078(63.8)	2084(67.5)	1002(32.5)	3086(63.8)
		Yes	1237(71.0)	506(29.0)	1743(35.2)	1201(68.6)	551(31.5)	1752(36.2)
	fishing	No	3353(70.1)	1428(29.9)	4781(99.2)	3266(68.1)	1532(31.9)	4798(99.2)
		Yes	31(77.5)	9(22.5)	40(0.8)	19(47.5)	21(52.5)	40(0.8)
	Government salaried	No	3163(70.4)	1330(29.6)	4493(93.2)	3063(67.9)	1446(32.1)	4509(93.2)
		Yes	221(67.4)	107(32.6)	328(6.8)	222(67.5)	107(32.5)	329(6.8)
	Private salaried	No	2998(71.1)	1220(28.9)	4218(87.5)	2898(68.5)	1331(31.5)	4229(87.4)
		Yes	386(64.0)	217(35.9)	603(12.5)	387(63.6)	222(36.5)	609(12.6)
	Informal salaried	No	3336(70.0)	1430(30.0)	4766(98.8)	3253(68.0)	1530(32.0)	4783(98.8)
		Yes	48(87.3)	7(12.7)	55(1.1)	32(58.2)	23(41.8)	55(1.1)
	Daily wage	No	3114(70.0)	1334(30.0)	4448(92.3)	3007(67.4)	1456(32.6)	4463(92.3)
		Yes	270(72.40	103(24.6)	373(7.7)	278(74.1)	97(25.9)	375(7.8)
	Bike ride	No	3141(70.1)	1340(29.9)	4481(92.9)	3040(67.6)	1457(32.4)	4497(92.9)
		Yes	243(71.5)	97(28.5)	340(7.1)	245(71.8)	96(28.2)	341(7.1)
	Stone mine	No	3271(70.0)	1401(30.0)	4672(96.9)	3161(67.4)	1528(32.6)	4689(96.9)
		Yes	113(75.8)	36(36.2)	149(3.1)	124(83.2)	25(16.8)	149(3.1)
	Unemployed	No	3378(70.2)	1435(29.8)	4813(99.8)	3279(67.9)	1551(32.1)	4830(99.8)
		Yes	6(75.0)	2(25.0)	8(0.2)	6(75.0)	2(25.0)	8(0.2)
	Others	No	3173(70.4)	1332(29.6)	4505(93.5)	3107(68.7)	1413(32.3)	4520(93.4)
		Yes	211(66.8)	105(33.2)	316(6.6)	178(56.0)	140(44.0)	318(6.6)
Total			3384(70.2)	1437(29.8)	4821(100.0)	3285(67.9)	1553(32.1)	4838(100.0)

Table 2 presents the estimated odds ratio (OR) and corresponding confidence intervals from the multilevel models analysing HU within the settlement and outside the settlement, highlighting the influence of various social, economic and environmental factors on healthcare access in these communities.

**Table 2:** Coefficient estimates (odds ratio, OR) and corresponding 95% credible intervals (95%CI) for healthcare utilisation (HU) within and outside informal settlements among households in Cockle Bay, Dwazark, and Moyiba

		HU within the informal settlement			HU outside the informal settlement		
		Model 1	Model 2	Model 3	Model 1	Model 2	Model 3
	Category: reference excluded	OR (95%CI)	OR (95%CI)	OR (95%CI)	OR (95%CI)	OR (95%CI)	OR (95%CI)
	Intercept	0.40(0.34, 0.47)	0.19(0.14, 0.27)	0.23(0.11, 0.47)	0.52(0.42, 0.64)	0.91(0.62, 1.36)	0.78(0.37, 1.66)
Head of household gender	Male		0.96(0.77, 1.21)	0.94(0.76, 1.17)		1.10(0.83, 1.45)	1.00(0.77, 1.30)
Disability in household	Yes		1.32(0.99, 1.76)	1.21(0.91, 1.60)*		2.01(1.43, 2.82) *	2.01(1.43, 2.83)*
Family type	Married/cohabit/engaged		1.05(0.78, 1.41)	1.00(0.75, 1.32)		1.30(0.90, 1.88)	1.23(0.86, 1.76)
	Divorced/separated/widowed		1.20(0.95, 1.49)	1.14(0.91, 1.40)		1.08(0.81, 1.45)	1.10(0.83, 1.46)
Income activity engagement	Yes		1.35(1.09, 1.70)	1.26(1.04, 1.56)*		1.22(0.93, 1.60)	1.26(0.97, 1.63)
Food security	Food insecure		1.32(1.06, 1.65)	1.22(0.98, 1.52)*		1.07(0.81, 1.42)	1.01(0.77, 1.32)
Community residence	Dwazark		2.62(2.03, 3.40)	4.31(2.57, 7.32)*		0.24(0.17, 0.33)	0.22(0.12, 0.38) *
	Moyiba		0.85(0.67, 1.10)	1.62(0.97, 2.75)*		0.21(0.15, 0.28)	0.22(0.13, 0.39)*
Household size				1.04(1.01, 1.07)			1.00(0.97, 1.02)
Length of residence	1–5 year			1.03(0.78, 1.37)			1.09(0.82, 1.44)
	6–10 year			1.15(0.86, 1.55)			1.08(0.81, 1.46)
	More than 10 years			1.43(1.08, 1.89*)			1.15(0.87, 1.53)
Household tenure	landlord			0.75(0.63, 0.89)*			1.09(0.92, 1.29)
	Free living			0.93(0.73, 1.18)*			1.07(0.84, 1.37)
	Caretaker/lease/ temporary stay/others			0.82(0.53, 1.24)			0.95(0.63, 1.43)
Piped dwelling	Yes			0.73(0.48, 1.09)			0.73(0.49, 1.07)
Piped neighbor	Yes			2.43(1.87, 3.18)*			0.99(0.76, 1.30)
Public tap/standpipe	Yes			0.88(0.72, 1.08)			0.85(0.69, 1.04)
Compound well	Yes			0.86(0.70, 1.07)			1.03(0.82, 1.29)
Spring water	Yes			1.19(0.97, 1.46)			1.06(0.87, 1.31)
Rainwater	Yes			0.87(0.74, 1.03)			0.64(0.54, 0.77)*
Bowser water	Yes			0.74(0.45, 1.18)			0.44(0.24, 0.79) *
Kiosk water	Yes			0.81(0.59, 1.10)			1.45(1.08, 1.94) *
Bottled water	Yes			0.45(0.23, 0.85)*			1.83(1.06, 3.16) *
Sachet water	Yes			0.74(0.64, 0.86)*			1.17(1.01, 1.37) *
Surface water	Yes			0.67(0.52, 0.86)*			0.64(0.48, 0.83) *
Neighbor’s well	Yes			1.27(1.04, 1.54)*			1.18(0.97, 1.43)
Other water sources	Yes			0.77(0.43, 1.35)			0.77(0.47, 1.28)
Water distance	30 min–1 h			0.77(0.64, 0.92)*			0.92(0.76, 1.11)
	1–2 h			0.60(0.47, 0.77)*			1.29(1.01, 1.63)*
	Over 2 h			0.57(0.46, 0.71)*			1.36(1.09, 1.70)
	Don’t know			1.18(0.77, 1.82)			
Water shortage	No			0.78(0.67, 0.91)*			0.81(0.69, 0.94)*
	Don’t know			0.73(0.20, 2.40)			
Flush	Yes			1.51(1.12, 2.04)*			1.60(1.21, 2.13) *
Latrine	Yes			1.14(0.84, 1.55)			0.86(0.72, 1.03)
Bucket	Yes			0.75(0.63, 0.88))*			1.38(0.97, 1.95)
Hanging	Yes			1.12(0.78, 1.60)			1.22(0.90, 1.64)
Open defecation	Yes			1.98(1.23, 3.19)*			2.41(1.46, 4.05) *
Other (toilet types)	Yes			1.04(0.43, 2.39)			0.79(0.33, 1.83)
Shared toilet	No			0.71(0.60, 0.84)*			1.01(0.85, 1.20)
	Not applicable			0.40(0.18, 0.83)*			
Around house	Yes			1.18(0.96, 1.46)			1.02(0.82, 1.28)
Dumping site	Yes			1.24(0.93, 1.63)			1.25(0.94, 1.67)
Drainage	Yes			1.18(0.99, 1.42)			0.90(0.75, 1.09)
Solid waste collectors	Yes			0.71(0.47, 1.06)			0.61(0.41, 0.93) *
Waste sea	Yes			1.35(0.84, 2.16)			1.09(0.66, 1.78)
Other (waste disposal)	Yes			1.52(1.07, 2.17) *			1.57(1.08, 2.26) *
Waste payment	No			1.16(0.79, 1.67)			1.47(1.01, 2.14) *
Environmental disaster	Yes			0.59(0.46, 0.75)*			0.78(0.60, 1.02)
business	Yes			1.08(0.92, 1.26)			1.28(1.09, 1.51) *
fishing	Yes			0.88(0.41, 1.79)			1.88(0.96, 3.68)
Government salaried	Yes			0.89(0.67, 1.16)			1.31(0.98, 1.74)
Private salaried	Yes			1.18(0.96, 1.45)			1.33(1.07, 1.65) *
Daily wage	Yes			0.88(0.68, 1.14)			0.97(0.74, 1.28)
Stone mine	Yes			1.02(0.67, 1.53)			0.54(0.33, 0.85) *
Other (Income sources)	Yes			1.08(0.82, 1.42)			1.55(1.17, 2.04) *
Study sample		4,821	4,821	4,821	4,616	4,616	4616
Strata number		122	122	122	122	122	122
Strata variance		0.39	0.06	0.03	0.73	0.18	0.13
VPC		10.49%	1.66%	0.84%	18.18%	5.23%	3.91%
PCV		-	85.56%	92.77%	-	75.18%	81.68%

Model 1: Null model; Model 2: main effects model; Model 3: main effects and explanatory variables model; OR (95%CI): Odds Ratio (95% Credible Interval); *: significant at 5% level; VPC: variance partitioning coefficient; PCV: proportional change in variance; reference categories excluded

### HU within the informal settlement

A total of 122 strata were constructed based on six factors with the strata sample sizes ranging from 1 to 551. Notably,48 (37.5%) strata have a sample size of fewer than 5 households. The description and sample size for each stratum are detailed in Supplementary Table 3a. The general contextual effect of the strata in the null model (Model 1), measured by the VPC, indicated that approximately 10.5% of the total variance in HU within is explained by the clustering of social strata. When the variables used to construct social strata were added as main effects in Model 2, the VPC reduced to 1.7%. The PCV of 85.6% suggests that most of the between-strata variance was explained by the inclusion of these the main effects, leaving 14.4% unexplained.

After adjusting for explanatory variables in Model 3, the strata level variance decreased further, resulting in a VPC of 0.9%. Approximately, 92.8% of the total variance between strata (PCV) was accounted for after including both the main effects and explanatory variables. This indicates that only 7.2% of the between strata differences in HU within the settlement can be attributed to social strata, suggesting the presence of moderate intersectional inequalities.

Model 3 shows that households with disabled members had significantly higher odds of HU within the settlement compared to those without disabilities. Households engaged in income-generating activities had 1.3 times higher odds of HU within settlement than those not engaged. The odds of HU within settlement were 4.3 times higher in Dwazark and 1.2 times higher in Moyiba compared to Cockle Bay. Households residing for 6–10 years or over 10 years had higher odds of HU within settlement compared to those residing for one year and less. There were no significant associations between HU within settlements and households’ sources. Additionally, landlords had significantly lower odds of HU within the settlement compared to tenants.

Households using piped water from neighbours, and neighbours well had 2.4, and 1.3 times higher odds of HU within settlement, respectively. In contrast, those relying on bottled water, sachet water, or surface water had lower odds. Households that spent 30 min to 1 h, 1–2 h, or more than 2 h fetching cooking water had significantly lower odds of HU compared to those taking less than 30 min. Households experiencing no water shortages also had lower odds of HU. Those using flush toilet and practising open defecation had 1.5 and 2.0 times higher odds of HU, respectively, while households using bucket toilets or not sharing toilets had lower odds. The odds of HU were higher among households using other waste disposal methods, whereas the rest waste disposal methods showed no significant associations. Finally, households that experienced environmental disasters had lower odds of HU within the settlement compared to those that did not.

**Fig. 1:**
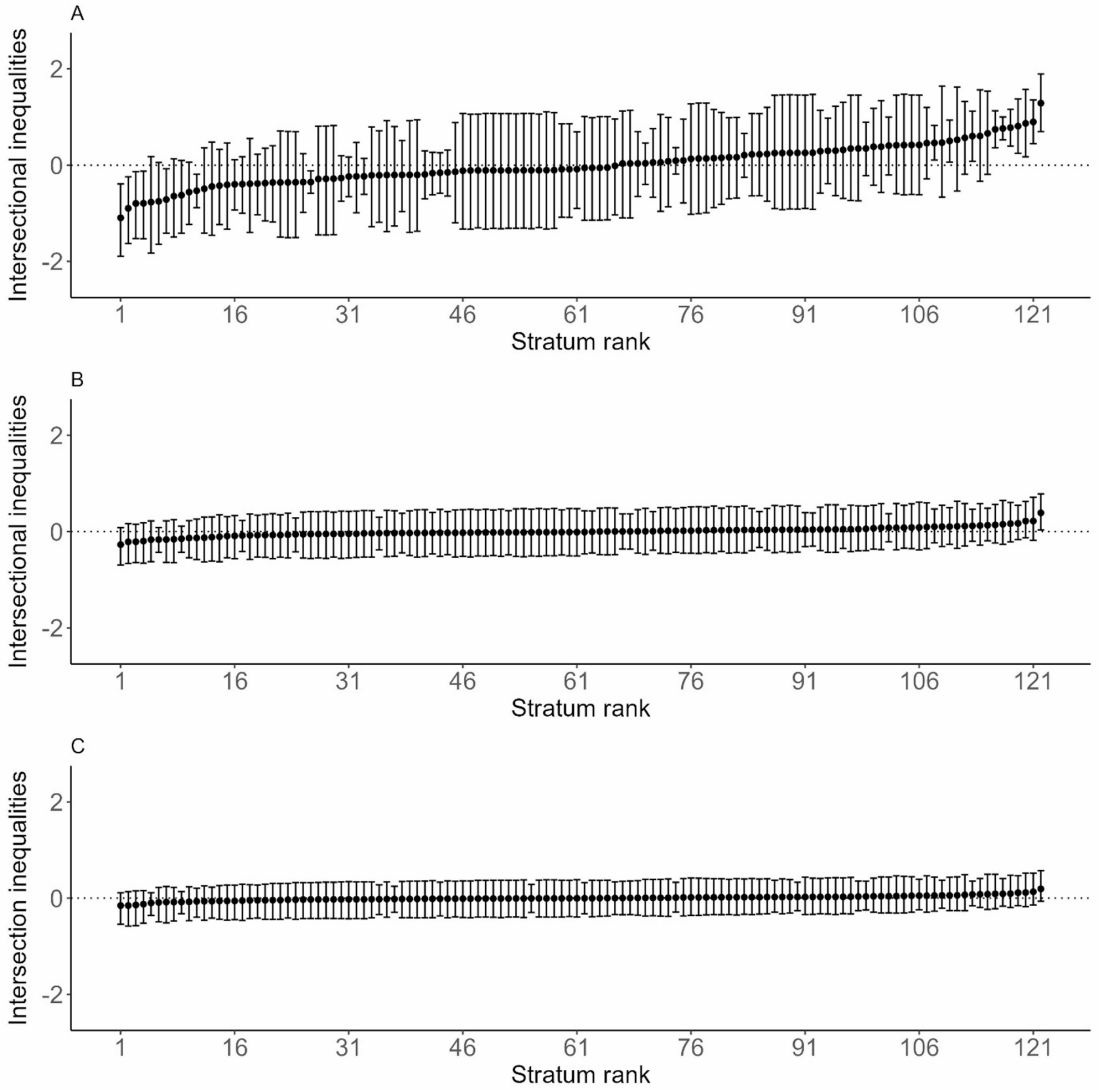
Estimated intersectional effect residual and their corresponding 95% credible intervals (CIs) for each stratum, ranked from the lowest to highest levels of HU within the informal settlements, are presented for Model 1 (Panel **A**), Model 2 (Panel **B**) and Model 3 (Panel **C**)

Figure 1 shows the strata residuals plotted in ascending order, illustrating the difference between the observed and expected HU probabilities within the settlement for each stratum. In Panel A, residuals ranged from − 1.1 to 1.3 in, with some strata exhibiting large confidence intervals due to small sample sizes. Several strata showed significant deviations from expected values based solely on strata random effects, indicating inequality in HU within the settlements. However, the inclusion of main effects (Panel B) and explanatory variables (Panel C) reduced the number of strata with significant intersectional effects.

Overall, single males with disabilities from Moyiba, who were food insecure and not engaged in income-generating activities, had the lowest levels of HU within the settlement. This was followed by single-female-headed households without a disabled member from Cockle Bay, who were food insecure and not actively engaged in income-generating activities. Additionally, female-headed households in Moyiba, whether food secure on insecure, single, without a disabled member and engaged in income generating activities, also exhibited low HU within the settlement.

The highest levels of HU within the settlement were observed among male-headed households in Dwazark that were married, cohabiting, or engaged; food insecure or secure, without a disabled household member, and actively involved in income-generating activities. This was followed by single female-headed households from Dwazark, whether food insecure or secure, without disabled member, and engaged in income-generating activities.

Overall, strata from Dwazark were highly concentrated at the upper end of HU within the settlement, while strata from Moyiba and Cockle Bay exhibited lower levels of HU. The results for food security were mixed, with food-secure and food-insecure households appearing in strata associated with both low and high HU within the settlement.

Posterior diagnostics measures, including the Rhat statistic, Bulk-ESS, and Tail-ESS indicated that Models 1, 2, and 3 achieved good convergence and sufficient sampling efficiency (Supplementary Tables 4a, 5a, and 6a, and Fig. 1a). The sensitivity analysis focusing on HU within settlements through formal through the formal healthcare providers within the settlements yielded results broadly consistent with those obtained when considering both formal and informal providers (Supplementary Table 7a). The VPC for HU in Models 1, 2, and 3 were 10.6%, 2.0%, and 1.1%, respectively. The PCV between Models 1 and 2 was 83.2%, and between Models 1 and 3, 90.3%. There was also consistency in the identification of strata at both the low and high levels of HU through formal healthcare providers within the settlements.

### HU outside the informal settlement

The sample size of the 122 strata ranged from 1 to 553, with 49 (38.3%) strata having a sample size of less than 5 households (Supplementary Table 3a).

The VPC in Model 1 was 18.2%, indicating a high level of clustering at the stratum-level. After the inclusion of the main effects in Model 2, the VPC decreased to 5.2%. The PCV for Model 2 was 75.2%, suggesting that 24.8% of the between strata-variance was attributable to intersection effects. The additional of explanatory variables in Model 3 further reduced the VPC to 3.9%. The PCV for Model 3 was 81.7%, indicating that 18.3% of the between-strata variance could still be attributed to intersectional effects, suggesting persistent and significant l inequalities in HU outside the informal settlements.

Model 3 shows that households with disabled members had twice the odds of HU outside the settlement compared to those without. Households in Dwazark and Moyiba had significantly lower odds of HU outside the settlement than those in Cockle Bay. Households with income from business, private salaried employment, or other sources had higher odds of HU outside the settlement compared to those without these income sources. In contrast, households engaged in stone mining had lower odds of HU outside the informal settlements.

Households using kiosk water, bottled water, and sachet water, had higher odds of HU outside the settlement 1.5, 1.9, and 1.2 times higher, respectively, compared to those not using these sources. In contrast, households that relied on rainwater and bowser water had lower odds of HU. Additionally, households that spent 1–2 h and more than 2 h to fetch cooking water had 1.3 times higher odds of HU outside the informal settlement compared to those who took less than 30 min.

Households using flush toilet and practising open defecation had higher odds of HU outside the settlement 1.6, and 2.4 times respectively. The odds of HU outside the settlement were lower for households using waste solid collectors compared to those not using them, while households disposing of waste in other locations had significantly higher odds (1.6 times). Additionally, households that paid for waste disposal had 1.5times higher odds of HU outside the settlement compared to those not paying.

**Fig. 2:**
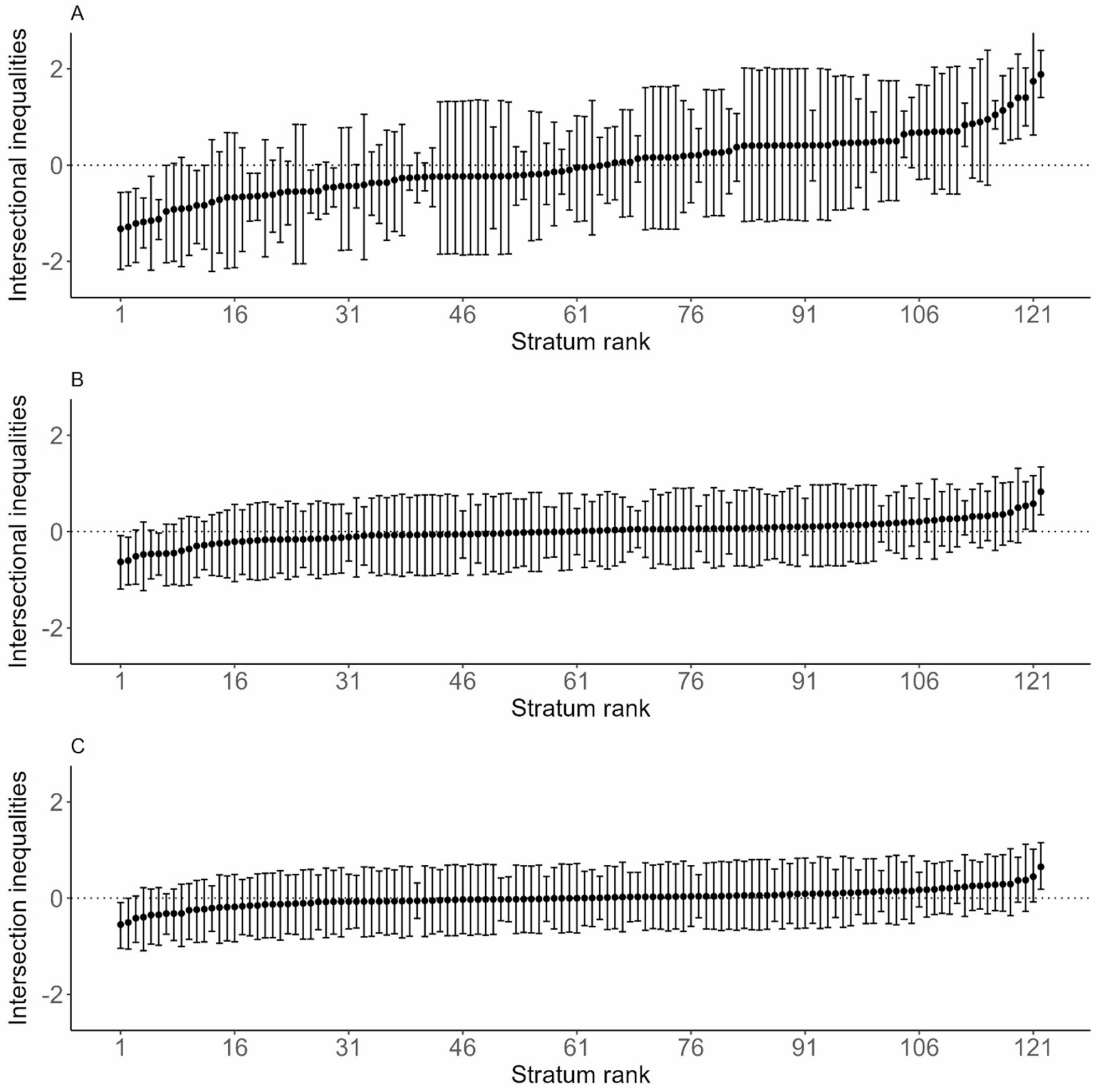
Estimated intersectional effect residual and their corresponding 95% credible intervals (CIs) for each stratum, ranked from the lowest to highest levels of HU outside the informal settlements, are presented for Model 1 (Panel **A**), Model 2 (Panel **B**) and Model 3 (Panel **C**)

Figure 2 displays the strata residuals, highlighting the difference between observed and expected HU probabilities outside the informal settlements for each group. Female-headed households from Moyiba, married, cohabiting, or engaged with a disabled household member, food insecure or secure, and engaged in income-generating activities had the lowest levels HU outside the settlement. They were followed by single, male-headed households from Moyiba with no income-generating activities and no disabled household member, as well as single female- headed households from Moyiba without a disabled member, food secure or insecure, and not engaged in income-generating activities.

Conversely, the highest levels of HU outside the informal settlement were observed among single female-headed households from Cockle Bay, without disabled household members and engaged in income-generating activities. This was followed by households from Cockle Bay with either male or female heads who were divorced, separated, or widowed, without disabled members and actively engaged in income-generating activities. Interestingly, single male-headed, households with a disabled person and engaged in income-generating activities from Cockle Bay also exhibited high HU outside settlement. Food security results were mixed, with both food-secure and food-insecure households appearing in strata with both low and high HU outside the settlement.

Posterior diagnostics measures for models examining HU outside the informal settlements indicated good convergence and sufficient sampling efficiency (Supplementary Tables 4a, 5a, and 6a, and Fig. 2a). Moreover, the sensitivity analysis for HU in formal healthcare providers outside the informal settlements yielded results broadly consistent with those obtained when considering both formal and informal providers (Supplementary Table 7a). The VPC for Models 1, 2, and 3 were 18.7%, 5.6%, and 3.9%, respectively. The PCV between Models 1 and 2 was 74.5%, and between Models 1 and 3 80.2%. There was also consistency in the identification of strata at both low and high levels of HU through formal providers outside the informal settlements.

The results for combined HU within and outside the informal settlements are presented in Supplementary Tables 8a and 9a, and Fig. 3a, showing a pattern similar to that of HU within and outside settlements separately (Table 1).

## Discussion

In this study, we applied an intersectional MAIHDA approach to examine HU inequalities among households in three informal settlements (i.e., Cockle Bay, Dwazark, and Moyiba) in Freetown, Sierra Leone. Our findings indicate that HU, both within and outside the informal settlements, is influenced by household-level social positions defined by gender and marital status of the household head, engagement in income-generating activities, food security status, disability status of household members, and settlement location. The PCV, after accounting for main effects and explanatory variables, indicated that 7.2% of the between strata-variance in HU within the informal settlement and 18.3% in HU outside the informal settlement was attributable to intersectional effects, highlighting moderate inequalities.

Key findings revealed that low HU within the informal settlement was observed among households headed by single, disabled males in Moyiba with food insecurity and not engaged in income-generating activities. In contrast, highest levels of HU within the settlement were observed among male-headed households in Dwazark that were married, cohabiting, or engaged; food insecure or secure, without a disabled household member, and actively involved in income-generating activities. In some cases, due to lack of public health facilities (e.g., Cockle Bay) [41] and difficulties accessing speciality care [42] within settlements, and concerns about privacy and stigma [38] households sought healthcare outside their informal settlements. The lowest levels of HU outside the informal settlement were observed among female-headed households from Moyiba, married, cohabiting, or engaged with a disabled household member, food insecure or secure, and engaged in income-generating activities s. Conversely, the highest HU outside the informal settlement was observed among male-headed households in Dwazark that were married, cohabiting, or engaged; food insecure or secure, without a disabled household member, and actively involved in income-generating activities.

These findings highlight moderate intersectional inequalities in HU, with lower levels of HU particularly evident among the most vulnerable groups. Vulnerable households in informal settlements are typically more prone to illnesses due to poor sanitation and unhealthy diets, and might therefore be expected to require more healthcare [52]. However, the data suggest that social strata composed of households with more positive socio-economic outlook tend to exhibit higher HU, both within and outside the informal settlements. This indicates that vulnerable households are marginalised in accessing healthcare, likely due to limited financial resources to support utilisation [9, 23, 38].

This aligns with previous research indicating that HU inequalities in informal urban areas are driven by factors such as poverty, unemployment, and socioeconomic status, all of which affect a households’ ability to afford healthcare [23, 43, 48]. This helps explains why social strata comprising households with single, disabled male-heads from Moyiba who were not engaged in income-generating activities, as well as divorced, separated, or widowed, female heads without income generating activities (both with and without disabled household members), had overall lower levels of HU on average compared to similar households in Cockle Bay and Dwazark that were engaged in income-generating activities. This is despite Cockle Bay lacking a formal healthcare facility [41]. Generally, intersectional strata from Moyiba showed lower levels of HU both within and outside informal settlements compared to those in Cockle Bay and Dwazark, as many respondents in Moyiba were engaged in low-wage jobs such as stone mining, fishing, bike riding and daily wage labour, unlike the other two settlements [41]. As a result, their limited ability to pay out-of-pocket for healthcare contributed to low HU [12, 21].

This underscores the need for stakeholders to develop interventions based on the principle of proportionate universalism, aimed at improving the overall HU among informal dwellers, with proportionate targeting of the most vulnerable households based on their level of need [13]. Proportionate universalism ensures that interventions are made available to everyone in the studied settlements (i.e., universal access), but with targeted intensity of support for identified vulnerable groups. For example, to reduce HU inequalities, it may be beneficial to introduce social security, health insurance, and outreach clinics for informal dwellers, initially prioritising households with no sources of income and headed by single men and women with disabled members. This recommendation is further supported by evidence showing that HU is higher among slum residents with healthcare insurance compared to those without [38].

Moreover, our findings highlight how the application of quantitative intersectionality helps reveal the interlocking systems of marginalisation and privilege in HU that can render certain households vulnerable, even when they might not appear so when viewed in isolation. For example, when gender is considered alone, female-headed households in urban slums typically have low HU compared to male-headed households [37]. However, our results show that due to the interlocking nature of marginalisation, certain single male-headed households with disabled members and no engagement in income-generating activities had lower HU both within and outside informal settlements than single-female-headed households, that were engaged in income-generating and did not have disabled members. It is important to note, however, that the social strata patterning of HU intersectional inequalities based on household food security was unclear. This may be due to the low sensitivity of the food security indicator used, which focused only on whether a household was able to eat the kinds of food they preferred; just one of the nine common experiences of food security [8, 41]. However, by engaging the community in the research process, we have gained a deeper understanding of HU inequalities in informal settlements, grounded in the lived experiences of residents. We further recommend that community members be actively involved in selecting social position variables.

Overall, we found that households characterised by protective factors against illnesses have higher HU, despite being less susceptible to illness, while those predisposed to illness-enabling factors generally exhibited lower HU. This disparity can be explained by that fact that households with protective factors are financially able to seek healthcare whenever needed, which is not the case for households facing greater vulnerability [38]. For example, households engaged in income-generating activities were associated with higher odds of HU, which aligns with findings from other studies, as most residents in informal settlements rely on out of pocket payments to access healthcare [12, 38]. In contrast, households with unreliable income sources, such as fishing, stone mining and daily wages, had lower HU, likely because they lack stable income during periods of illness, which negatively impacts their ability to seek care. Additionally, households using flush toilets and clean water sources, such as compound well, bottled water, and neighbours wells, had higher odds of HU, as they are generally more financially better off and able to afford healthcare [38].

This study has some limitations. First, it assumes that the social factors identified by community members fully capture the dimensions of social positions at household level that influence HU. However, these social factors should be interpreted in the specific context of the studied population and outcome, as interlocking systems of power and oppression may differ in other settings. Second, our analyses were conducted at the household level, and we did not account for individual factors such as age, gender, ethnicity, and lifestyle choices which also play a significant role in influencing HU in informal settlements. Third, the study was limited to data from only three informal settlements, which may affect the generalisability of the results to other informal urban areas in Freetown.

Fourth, since HU was assessed during the dry season, our findings may not fully capture seasonal variability in HU. The absence of rainy-season stressors such as flooding and cholera outbreaks in Cockle Bay, and poor accessibility to health facilities during the rainy season in the hilly terrain of Moyiba, means these seasonal challenges were not reflected in the data. Additionally, the individual interviewed may not accurately recall healthcare services sought by all household members within the one-month window. Misreporting may also occur when some members choose not to disclose their healthcare use due to social desirability; particularly when the condition is stigmatised, such as sexually transmitted infections.

Fifth, we acknowledge that the definition of HU used in this study does not account for unmet need [29]. In addition, we did not control for household size in Models 1 and 2 to maintain alignment with the MAIHDA framework. Generally, larger households are more likely to seek healthcare services. However, household size was only significant for HU within the settlement in Model 3, and not for HU outside, indicating its limited overall influence. Finally, the high number of social strata with small sample sizes (fewer than five households) particularly those with disabled persons may have obscured the detection of some intersection effects [15, 34].

In conclusion, despite these limitations, we have identified and quantified moderate intersectional inequalities in HU across three informal settlements in Freetown, Sierra Leone; for the first time. Future studies should explore whether these inequalities vary according to social positions defined by individual-level factors.

## Supplementary Information

Below is the link to the electronic supplementary material.

Supplementary Material 1

## Data Availability

The data used in this study include sensitive category household-level data. To prevent disclosure these data are not publicly available but are available for research purposes through successful application to data holders (Liverpool School of Tropical Medicine, College of Medicine and Allied Health Sciences (COMAHS) - University of Sierra Leone, Sierra Leone Urban Research Centre (SLURC), and Centre of Dialogue on Human Settlement and Poverty Alleviation (CODOHSAPA)-Sierra Leone).
