# Supplementary material for: Intersectional inequalities in healthcare utilisation in informal settlements in Freetown, Sierra Leone: a multilevel analysis of individual heterogeneity and discriminatory accuracy (MAIHDA)
Source: Int J Equity Health. 2026 Apr 14;25:131. doi: 10.1186/s12939-026-02851-w (PMC13185398; doi:10.1186/s12939-026-02851-w)
Supplement: Supplementary file 1 — Supplementary Material 1 [file 12939_2026_2851_MOESM1_ESM.docx]

**Supplementary: Intersectional inequalities in healthcare utilisation in informal settlements in Freetown Sierra Leone. A multilevel analysis of individual heterogeneity and discriminatory accuracy (MAIHDA).**

Table 1a: Definition of variables

|  | **Definition** |
| --- | --- |
| **Health utilisation** | Did you or any member of your household seek health provision service during the last one month within your community? |
| **Disability** | Are there individuals with disabilities in this household? |
| **Family type** |  |
| Single | Unmarried or not involved in a stable relationship |
| Married/cohabiting/engaged | Legally and socially sanctioned union including cohabiting and engaged |
| Divorced/Separated/widowed | No longer married or marriage legally dissolved or having lost a husband or wife through death |
| **Income activity** | Are there any household member engaged in any form of income activity like business |
| **Household tenure** |  |
| Tenant | Households who pay house rent to the landlords |
| Landlord | People who own the house they live in |
| Free-living | They neither pay rent or own the house |
| Others | Caretaker, lease, temporal stay |
| **Household income** |  |
| Business | Households in any form of buying and selling as a source of income. |
| Government salaried | Households in any government paid jobs. |
| Private salaried | Households in any form of private paid jobs. |
| Informal salaried | Households engaged in not formal paid jobs e.g. nanny, cleaner sex workers |
| Daily wage | Labourers earning on a daily |
| Bike riders | Drivers of motorbikes as transportation in hard to reach areas |
| Stone mine | Individuals who manually mine/crush stone and sell them |
| Unemployed | No jobs that provide income |
| Others | Babysitter, farmer, footballer, hair dresser, plumber, tailoring etc |
| **Food security** |  |
| Food secure | Any household member able to eat the kinds of food they preferred due to affordability of resources |
| Food insecure | Any household member not able to eat the kinds of food the preferred due to a lack of resources |
| **Length of Stay** | How long household members have stayed within the community |
| **Water source** |  |
| Kiosk/bowsers | Large water containers in strategic areas, protected and managed by community members and sometimes paid for by users |
| bottled | Private water vendors in bottles for high income users |
| sachet | Private water vendors in plastic packages for low income users |
| surface | Water that licks out anywhere from the ground below and most times not protected |
| other | Stream, borehole |
| **Toilet type** |  |
| Flush | A more improved/advanced toilet facility for proper sanitation |
| Latrine | A simple hole/trench in the ground |
| Bucket | Small portable buckets for faeces |
| Hanging | Form of toilets built over water body |
| Flying | Defecation in plastic bags and then thrown |
| Open defecation | Practice of defecating outside like bushes, canals streams |
| other | Public toilets, plastic bags, in the sea |
| **Waste disposal** |  |
| Around house | Just throw waste somewhere/anywhere around the house/compound |
| Dumping site | In a designated waste area |
| Solid collectors | Individuals or institutions paid for collecting waste in houses |
| other | burning |

Table 3a: Strata categories and number of households in each stratum

| Strata Categories:  **Gender, marital status, income activity, disability, food security, community** | Number of households in each stratum | | |
| --- | --- | --- | --- |
|  | HU within settlement | HU outside settlement | HU within and outside settlement |
| Female, Divorced_Separated_Widowed, No, No, food_insecure, Cocklebay | 12 | 12 | 12 |
| Female, Divorced_Separated_Widowed, No, No, food_insecure, Dwazark | 38 | 38 | 38 |
| Female, Divorced_Separated_Widowed, No, No, food_insecure, Moyiba | 19 | 19 | 19 |
| Female, Divorced_Separated_Widowed, No, No, food_secure, Cocklebay | 6 | 6 | 6 |
| Female, Divorced_Separated_Widowed, No, No, food_secure, Dwazark | 7 | 7 | 7 |
| Female, Divorced_Separated_Widowed, No, No, food_secure, Moyiba | 11 | 11 | 11 |
| Female, Divorced_Separated_Widowed, No, Yes, food_insecure, Dwazark | 5 | 5 | 5 |
| Female, Divorced_Separated_Widowed, No, Yes, food_secure, Dwazark | 1 | 1 | 1 |
| Female, Divorced_Separated_Widowed, No, Yes, food_secure, Moyiba | 1 | 1 | 1 |
| Female, Divorced_Separated_Widowed, Yes, No, food_insecure, Cocklebay | 38 | 38 | 38 |
| Female, Divorced_Separated_Widowed, Yes, No, food_insecure, Dwazark | 84 | 84 | 84 |
| Female, Divorced_Separated_Widowed, Yes, No, food_insecure, Moyiba | 60 | 60 | 60 |
| Female, Divorced_Separated_Widowed, Yes, No, food_secure, Cocklebay | 21 | 21 | 21 |
| Female, Divorced_Separated_Widowed, Yes, No, food_secure, Dwazark | 38 | 38 | 38 |
| Female, Divorced_Separated_Widowed, Yes, No, food_secure, Moyiba | 86 | 86 | 86 |
| Female, Divorced_Separated_Widowed, Yes, Yes, food_insecure, Cocklebay | 3 | 3 | 3 |
| Female, Divorced_Separated_Widowed, Yes, Yes, food_insecure, Dwazark | 9 | 9 | 9 |
| Female, Divorced_Separated_Widowed, Yes, Yes, food_insecure, Moyiba | 8 | 8 | 8 |
| Female, Divorced_Separated_Widowed, Yes, Yes, food_secure, Cocklebay | 2 | 2 | 2 |
| Female, Divorced_Separated_Widowed, Yes, Yes, food_secure, Dwazark | 3 | 3 | 3 |
| Female, Divorced_Separated_Widowed, Yes, Yes, food_secure, Moyiba | 9 | 9 | 9 |
| Female, Married_cohabit_engaged, No, No, food_insecure, Cocklebay | 5 | 5 | 5 |
| Female, Married_cohabit_engaged, No, No, food_insecure, Dwazark | 7 | 7 | 7 |
| Female, Married_cohabit_engaged, No, No, food_insecure, Moyiba | 10 | 10 | 10 |
| Female, Married_cohabit_engaged, No, No, food_secure, Cocklebay | 6 | 7 | 7 |
| Female, Married_cohabit_engaged, No, No, food_secure, Dwazark | 4 | 4 | 4 |
| Female, Married_cohabit_engaged, No, No, food_secure, Moyiba | 24 | 24 | 24 |
| Female, Married_cohabit_engaged, No, Yes, food_insecure, Dwazark | 2 | 2 | 2 |
| Female, Married_cohabit_engaged, No, Yes, food_secure, Cocklebay | 2 | 2 | 2 |
| Female, Married_cohabit_engaged, No, Yes, food_secure, Moyiba | 2 | 2 | 2 |
| Female, Married_cohabit_engaged, Yes, No, food_insecure, Cocklebay | 16 | 16 | 16 |
| Female, Married_cohabit_engaged, Yes, No, food_insecure, Dwazark | 37 | 37 | 37 |
| Female, Married_cohabit_engaged, Yes, No, food_insecure, Moyiba | 49 | 49 | 49 |
| Female, Married_cohabit_engaged, Yes, No, food_secure, Cocklebay | 22 | 22 | 22 |
| Female, Married_cohabit_engaged, Yes, No, food_secure, Dwazark | 20 | 20 | 20 |
| Female, Married_cohabit_engaged, Yes, No, food_secure, Moyiba | 138 | 139 | 139 |
| Female, Married_cohabit_engaged, Yes, Yes, food_insecure, Dwazark | 4 | 4 | 4 |
| Female, Married_cohabit_engaged, Yes, Yes, food_insecure, Moyiba | 7 | 7 | 7 |
| Female, Married_cohabit_engaged, Yes, Yes, food_secure, Cocklebay | 3 | 3 | 3 |
| Female, Married_cohabit_engaged, Yes, Yes, food_secure, Dwazark | 1 | 1 | 1 |
| Female, Married_cohabit_engaged, Yes, Yes, food_secure, Moyiba | 12 | 12 | 12 |
| Female, Single, No, No, food_insecure, Cocklebay | 23 | 23 | 23 |
| Female, Single, No, No, food_insecure, Dwazark | 23 | 23 | 23 |
| Female, Single, No, No, food_insecure, Moyiba | 26 | 26 | 26 |
| Female, Single, No, No, food_secure, Cocklebay | 36 | 36 | 36 |
| Female, Single, No, No, food_secure, Dwazark | 23 | 23 | 23 |
| Female, Single, No, No, food_secure, Moyiba | 47 | 47 | 47 |
| Female, Single, No, Yes, food_insecure, Dwazark | 4 | 4 | 4 |
| Female, Single, No, Yes, food_insecure, Moyiba | 1 | 1 | 1 |
| Female, Single, No, Yes, food_secure, Cocklebay | 1 | 1 | 1 |
| Female, Single, No, Yes, food_secure, Dwazark | 2 | 2 | 2 |
| Female, Single, No, Yes, food_secure, Moyiba | 1 | 1 | 1 |
| Female, Single, Yes, No, food_insecure, Cocklebay | 40 | 40 | 40 |
| Female, Single, Yes, No, food_insecure, Dwazark | 64 | 64 | 64 |
| Female, Single, Yes, No, food_insecure, Moyiba | 40 | 40 | 40 |
| Female, Single, Yes, No, food_secure, Cocklebay | 61 | 63 | 63 |
| Female, Single, Yes, No, food_secure, Dwazark | 24 | 24 | 24 |
| Female, Single, Yes, No, food_secure, Moyiba | 62 | 62 | 62 |
| Female, Single, Yes, Yes, food_insecure, Cocklebay | 4 | 4 | 4 |
| Female, Single, Yes, Yes, food_insecure, Dwazark | 5 | 5 | 5 |
| Female, Single, Yes, Yes, food_insecure, Moyiba | 1 | 1 | 1 |
| Female, Single, Yes, Yes, food_secure, Cocklebay | 3 | 3 | 3 |
| Female, Single, Yes, Yes, food_secure, Dwazark | 1 | 1 | 1 |
| Female, Single, Yes, Yes, food_secure, Moyiba | 2 | 2 | 2 |
| Male, Divorced_Separated_Widowed, No, No, food_insecure, Cocklebay | 2 | 2 | 2 |
| Male, Divorced_Separated_Widowed, No, No, food_insecure, Dwazark | 5 | 5 | 5 |
| Male, Divorced_Separated_Widowed, No, No, food_insecure, Moyiba | 3 | 3 | 3 |
| Male, Divorced_Separated_Widowed, No, No, food_secure, Cocklebay | 1 | 1 | 1 |
| Male, Divorced_Separated_Widowed, No, No, food_secure, Dwazark | 2 | 2 | 2 |
| Male, Divorced_Separated_Widowed, No, No, food_secure, Moyiba | 9 | 9 | 9 |
| Male, Divorced_Separated_Widowed, No, Yes, food_secure, Dwazark | 1 | 1 | 1 |
| Male, Divorced_Separated_Widowed, Yes, No, food_insecure, Cocklebay | 4 | 4 | 4 |
| Male, Divorced_Separated_Widowed, Yes, No, food_insecure, Dwazark | 17 | 17 | 17 |
| Male, Divorced_Separated_Widowed, Yes, No, food_insecure, Moyiba | 20 | 20 | 20 |
| Male, Divorced_Separated_Widowed, Yes, No, food_secure, Cocklebay | 6 | 6 | 6 |
| Male, Divorced_Separated_Widowed, Yes, No, food_secure, Dwazark | 7 | 7 | 7 |
| Male, Divorced_Separated_Widowed, Yes, No, food_secure, Moyiba | 14 | 14 | 14 |
| Male, Divorced_Separated_Widowed, Yes, Yes, food_insecure, Dwazark | 2 | 2 | 2 |
| Male, Divorced_Separated_Widowed, Yes, Yes, food_insecure, Moyiba | 1 | 1 | 1 |
| Male, Divorced_Separated_Widowed, Yes, Yes, food_secure, Dwazark | 2 | 2 | 2 |
| Male, Divorced_Separated_Widowed, Yes, Yes, food_secure, Moyiba | 1 | 1 | 1 |
| Male, Married_cohabit_engaged, No, No, food_insecure, Cocklebay | 58 | 58 | 58 |
| Male, Married_cohabit_engaged, No, No, food_insecure, Dwazark | 98 | 98 | 98 |
| Male, Married_cohabit_engaged, No, No, food_insecure, Moyiba | 94 | 94 | 94 |
| Male, Married_cohabit_engaged, No, No, food_secure, Cocklebay | 65 | 65 | 65 |
| Male, Married_cohabit_engaged, No, No, food_secure, Dwazark | 44 | 44 | 44 |
| Male, Married_cohabit_engaged, No, No, food_secure, Moyiba | 115 | 117 | 117 |
| Male, Married_cohabit_engaged, No, Yes, food_insecure, Cocklebay | 3 | 3 | 3 |
| Male, Married_cohabit_engaged, No, Yes, food_insecure, Dwazark | 10 | 11 | 11 |
| Male, Married_cohabit_engaged, No, Yes, food_insecure, Moyiba | 5 | 5 | 5 |
| Male, Married_cohabit_engaged, No, Yes, food_secure, Cocklebay | 2 | 2 | 2 |
| Male, Married_cohabit_engaged, No, Yes, food_secure, Dwazark | 3 | 3 | 3 |
| Male, Married_cohabit_engaged, No, Yes, food_secure, Moyiba | 8 | 8 | 8 |
| Male, Married_cohabit_engaged, Yes, No, food_insecure, Cocklebay | 217 | 217 | 217 |
| Male, Married_cohabit_engaged, Yes, No, food_insecure, Dwazark | 362 | 362 | 362 |
| Male, Married_cohabit_engaged, Yes, No, food_insecure, Moyiba | 466 | 467 | 467 |
| Male, Married_cohabit_engaged, Yes, No, food_secure, Cocklebay | 229 | 229 | 229 |
| Male, Married_cohabit_engaged, Yes, No, food_secure, Dwazark | 209 | 209 | 209 |
| Male, Married_cohabit_engaged, Yes, No, food_secure, Moyiba | 551 | 553 | 553 |
| Male, Married_cohabit_engaged, Yes, Yes, food_insecure, Cocklebay | 14 | 14 | 14 |
| Male, Married_cohabit_engaged, Yes, Yes, food_insecure, Dwazark | 34 | 34 | 34 |
| Male, Married_cohabit_engaged, Yes, Yes, food_insecure, Moyiba | 39 | 39 | 39 |
| Male, Married_cohabit_engaged, Yes, Yes, food_secure, Cocklebay | 8 | 8 | 8 |
| Male, Married_cohabit_engaged, Yes, Yes, food_secure, Dwazark | 13 | 13 | 13 |
| Male, Married_cohabit_engaged, Yes, Yes, food_secure, Moyiba | 31 | 31 | 31 |
| Male, Single, No, No, food_insecure, Cocklebay | 24 | 25 | 25 |
| Male, Single, No, No, food_insecure, Dwazark | 38 | 38 | 38 |
| Male, Single, No, No, food_insecure, Moyiba | 61 | 62 | 62 |
| Male, Single, No, No, food_secure, Cocklebay | 50 | 50 | 50 |
| Male, Single, No, No, food_secure, Dwazark | 31 | 31 | 31 |
| Male, Single, No, No, food_secure, Moyiba | 87 | 87 | 87 |
| Male, Single, No, Yes, food_insecure, Cocklebay | 2 | 2 | 2 |
| Male, Single, No, Yes, food_insecure, Dwazark | 2 | 2 | 2 |
| Male, Single, No, Yes, food_insecure, Moyiba | 7 | 7 | 7 |
| Male, Single, No, Yes, food_secure, Cocklebay | 1 | 1 | 1 |
| Male, Single, No, Yes, food_secure, Moyiba | 4 | 4 | 4 |
| Male, Single, Yes, No, food_insecure, Cocklebay | 49 | 49 | 49 |
| Male, Single, Yes, No, food_insecure, Dwazark | 60 | 60 | 60 |
| Male, Single, Yes, No, food_insecure, Moyiba | 71 | 74 | 74 |
| Male, Single, Yes, No, food_secure, Cocklebay | 64 | 64 | 64 |
| Male, Single, Yes, No, food_secure, Dwazark | 36 | 36 | 36 |
| Male, Single, Yes, No, food_secure, Moyiba | 107 | 109 | 109 |
| Male, Single, Yes, Yes, food_insecure, Cocklebay | 4 | 4 | 4 |
| Male, Single, Yes, Yes, food_insecure, Dwazark | 6 | 6 | 6 |
| Male, Single, Yes, Yes, food_insecure, Moyiba | 5 | 5 | 5 |
| Male, Single, Yes, Yes, food_secure, Cocklebay | 3 | 3 | 3 |
| Male, Single, Yes, Yes, food_secure, Dwazark | 1 | 1 | 1 |
| Male, Single, Yes, Yes, food_secure, Moyiba | 7 | 7 | 7 |
|  |  |  |  |

Table 4a: Diagnostic measures assessing the quality of posterior estimates for HU within settlement and HU outside informal settlements in Model 1: Rhat, Bulk effective sample size (ESS) and tail ESS.

|  |  | HU within the settlement | | | HU outside the settlement | | |
| --- | --- | --- | --- | --- | --- | --- | --- |
|  |  | Rhat | Bulk ESS | Tail ESS | Rhat | Bulk ESS | Tail ESS |
|  | Intercept | 1.00 | 8040.20 | 15480.40 | 1.00 | 6702.21 | 12752.11 |
| Random intercept | | 1.00 | 10232.65 | 17730.19 | 1.00 | 8705.60 | 16796.60 |

Model 1: Null model

Table 5a: Diagnostic measures assessing the quality of posterior estimates for HU within settlement and HU outside informal settlements in Model 2: Rhat, Bulk effective sample size (ESS) and tail ESS.

|  |  | HU within the settlement | | | HU outside the settlement | | |
| --- | --- | --- | --- | --- | --- | --- | --- |
|  |  | Rhat | Bulk ESS | Tail ESS | Rhat | Bulk ESS | Tail ESS |
|  | Intercept | 1.00 | 20258.22 | 21676.65 | 1.00 | 11482.14 | 18428.99 |
| Head of household | Male | 1.00 | 29446.79 | 26669.5 | 1.00 | 18666.52 | 30052.40 |
| Disability in household | Yes | 1.00 | 34667.25 | 27565.57 | 1.00 | 42730.62 | 23729.17 |
| Family type | Married/cohabit/engaged | 1.00 | 34052.16 | 27154.93 | 1.00 | 19338.68 | 23449.06 |
|  | Divorced/separated/widowed | 1.00 | 26179.17 | 26434.12 | 1.00 | 15454.13 | 21654.19 |
| Income activity engagement | Yes | 1.00 | 26842.81 | 25931.09 | 1.00 | 18255.36 | 23385.29 |
| Food security | Food insecure | 1.00 | 31012.67 | 26656.43 | 1.00 | 17708.16 | 22632.09 |
| Community residence | Dwazark | 1.00 | 24794.88 | 26681.07 | 1.00 | 15487.65 | 21277.38 |
|  | Moyiba | 1.00 | 25381.53 | 27296.05 | 1.00 | 14319.85 | 21339.91 |
| Random intercept | | 1.00 | 8391.60 | 14563.7 | 1.00 | 13324.65 | 21768.83 |

Model 2: main effects model variables

Table 6a: Diagnostic measures assessing the quality of posterior estimates for HU within settlement and HU outside informal settlements in model 3: Rhat, Bulk effective sample size (ESS) and tail ESS.

|  |  | HU within the settlement | | | HU outside the settlement | | |
| --- | --- | --- | --- | --- | --- | --- | --- |
|  |  | Rhat | Bulk ESS | Tail ESS | Rhat | Bulk ESS | Tail ESS |
|  | Intercept | 1.00 | 35216.31 | 28180.33 | 1.00 | 23293.95 | 26464.32 |
| Head of household | Male | 1.00 | 41276.28 | 28619.92 | 1.00 | 39398.37 | 30052.4 |
| Disability in household | Yes | 1.00 | 65364.43 | 28049.00 | 1.00 | 42730.62 | 30299.1 |
| Family type | Married/cohabit/engaged | 1.00 | 52145.36 | 28863.72 | 1.00 | 40269.52 | 30401.94 |
|  | Divorced/separated/widowed | 1.00 | 43589.23 | 29109.56 | 1.00 | 30771.62 | 27965.91 |
| Income activity engagement | Yes | 1.00 | 34960.85 | 27903.99 | 1.00 | 35068.36 | 30220.01 |
| Food security | Food insecure | 1.00 | 48695.79 | 29251.66 | 1.00 | 35877.35 | 30252.16 |
| Community residence | Dwazark | 1.00 | 27735.69 | 26681.07 | 1.00 | 35241.61 | 28520.76 |
|  | Moyiba | 1.00 | 29640.51 | 27296.05 | 1.00 | 34363.14 | 29678.47 |
| Household size |  | 1.00 | 41276.28 | 29112.07 | 1.00 | 72304.65 | 27818.04 |
| Length of residence | 1-5 year | 1.00 | 33714.54 | 28352.34 | 1.00 | 36947.58 | 29620.15 |
|  | 6-10 year | 1.00 | 32806.33 | 29665 | 1.00 | 35063.26 | 29989.19 |
|  | More than 10 years | 1.00 | 32072.3 | 27837.3 | 1.00 | 35804.74 | 31169.18 |
| Household tenure | landlord | 1.00 | 64805.67 | 29395.87 | 1.00 | 64792.29 | 29468.62 |
|  | Free living | 1.00 | 79118.62 | 27932.91 | 1.00 | 74095.98 | 26995.65 |
|  | Caretaker/lease/  temporary stay/others | 1.00 | 84328.05 | 24875.24 | 1.00 | 85623.39 | 25955.73 |
| Piped dwelling | Yes | 1.00 | 78067.15 | 27092.82 | 1.00 | 82440.45 | 26779.7 |
| Piped neighbor | Yes | 1.00 | 83660.26 | 27152.63 | 1.00 | 80151.49 | 26878.93 |
| Public tap/standpipe | Yes | 1.00 | 58570.89 | 30589.64 | 1.00 | 57860.38 | 29653.43 |
| Compound well | Yes | 1.00 | 59938.1 | 30559.33 | 1.00 | 64616.76 | 28679.37 |
| Spring water | Yes | 1.00 | 55800.96 | 29391.69 | 1.00 | 55345.22 | 29812.78 |
| Rainwater | Yes | 1.00 | 50512.78 | 29101.17 | 1.00 | 54089.23 | 30734.9 |
| Bowser water | Yes | 1.00 | 82927.86 | 25493.52 | 1.00 | 86820.96 | 26186.07 |
| Kiosk water | Yes | 1.00 | 73619.22 | 26817.11 | 1.00 | 80966.49 | 26607.26 |
| Bottled water | Yes | 1.00 | 87651.43 | 24978.91 | 1.00 | 89942.46 | 25686.82 |
| Sachet water | Yes | 1.00 | 82081.08 | 26771.25 | 1.00 | 83152.95 | 26631.1 |
| Surface water | Yes | 1.00 | 72283.18 | 27983.76 | 1.00 | 73260.75 | 26398.95 |
| Neighbor’s well | Yes | 1.00 | 61435.66 | 27707.98 | 1.00 | 66120.33 | 29000.13 |
| Other water sources | Yes | 1.00 | 80800.17 | 26390.62 | 1.00 | 83319.63 | 26302.19 |
| Water distance | 30 minutes-1 hours | 1.00 | 47017.57 | 31546.65 | 1.00 | 51522.27 | 31480.39 |
|  | 1- 2 hours | 1.00 | 48155.24 | 29883.85 | 1.00 | 48256.67 | 30458.06 |
|  | Over 2 hours | 1.00 | 43300.32 | 32574.47 | 1.00 | 46063.26 | 31368.03 |
|  | Don’t know | 1.00 | 61629.17 | 28424.51 | 1.00 |  |  |
| Water shortage | No | 1.00 | 69090.74 | 29358.78 | 1.00 | 77100.98 | 27909.88 |
|  | Don’t know | 1.00 | 83367.39 | 24921.25 | 1.00 |  |  |
| Flush | Yes | 1.00 | 36361.78 | 31421.52 | 1.00 | 38508.37 | 31356.9 |
| Latrine | Yes | 1.00 | 68498.31 | 28312.86 | 1.00 | 63009 | 28470.67 |
| Bucket | Yes | 1.00 | 47802.02 | 32345.34 | 1.00 | 46024.61 | 31477.84 |
| Hanging | Yes | 1.00 | 36387.53 | 30916.67 | 1.00 | 35327.37 | 29884.05 |
| Open defecation | Yes | 1.00 | 66156.91 | 26976.52 | 1.00 | 72768.84 | 26835.84 |
| Other (toilet types) | Yes | 1.00 | 73331.6 | 27041.04 | 1.00 | 71964.65 | 25702.13 |
| Shared toilet | No | 1.00 | 77413.07 | 27892.67 | 1.00 | 70623.7 | 26825.58 |
|  | Not applicable | 1.00 | 86395.89 | 26657.64 | 1.00 | 59371.73 |  |
| Around house | Yes | 1.00 | 55271.61 | 31030.47 | 1.00 | 62118.47 | 30898.59 |
| Dumping site | Yes | 1.00 | 67995.33 | 28582.23 | 1.00 | 63503.93 | 28967.47 |
| Drainage | Yes | 1.00 | 59844.22 | 29643.87 | 1.00 | 44848.88 | 27830.69 |
| Solid waste collectors | Yes | 1.00 | 44109.2 | 27780.54 | 1.00 | 42980.67 | 29528.62 |
| Waste sea | Yes | 1.00 | 32909.9 | 28579.6 | 1.00 | 67573.84 | 29187.68 |
| Other (waste disposal) | Yes | 1.00 | 68321.5 | 29262.31 | 1.00 | 48768.46 | 29209.14 |
| Waste payment | No | 1.00 | 47739.71 | 27176.21 | 1.00 | 83958.58 | 29365.22 |
| Environmental disaster | Yes | 1.00 | 80796.76 | 26862.67 | 1.00 | 61395.67 | 25730.31 |
| business | Yes | 1.00 | 62289.59 | 29673.63 | 1.00 | 91708.98 | 30627.68 |
| fishing | Yes | 1.00 | 88875.55 | 24168.88 | 1.00 | 76773.26 | 25276.94 |
| Government salaried | Yes | 1.00 | 73022.12 | 27715.17 | 1.00 | 68332.85 | 29366.14 |
| Private salaried | Yes | 1.00 | 71593.88 | 30554.74 | 1.00 | 77847.27 | 29162.3 |
| Daily wage | Yes | 1.00 | 70916.87 | 26763.8 | 1.00 | 83460.84 | 27763.17 |
| Stone mine | Yes | 1.00 | 79998.89 | 26457.1 | 1.00 | 69178.04 | 25756.59 |
| Other (Income sources) | Yes | 1.00 | 68362.11 | 28528.23 | 1.00 | 15267.01 | 30353.01 |
| Random intercept | | 1.00 | 8391.60 | 11185.81 | 1.00 | 38508.37 | 22217.09 |

Model 3: main effects and explanatory variables model

Figure 1a: Trace plot showing sampled values of the standard deviation and posterior density plot showing the distribution of estimated values for the random intercept (intersectional strata) in health utilisation within informal settlements (Model 3)

Figure 2a: Trace plot showing sampled values of the standard deviation and posterior density plot showing the distribution of estimated values for the random intercept (intersectional strata) in health utilisation outside informal settlements (Model 3)

Table 7a: Coefficient estimates (odds ratio, OR) and corresponding 95% credible intervals (95%CI) for formal healthcare utilisation (HU) within and outside settlements among households of Cockle Bay, Dwazark, and Moyiba

|  |  | HU within the settlement | | | HU outside the settlement | | |
| --- | --- | --- | --- | --- | --- | --- | --- |
|  |  | Model 1 | Model 2 | Model 3 | Model 1 | Model 2 | Model 3 |
|  | Category: reference excluded | OR (95%CI) | OR (95%CI) | OR (95%CI) | OR (95%CI) | OR (95%CI) | OR (95%CI) |
|  | Intercept | 0.27(0.23, 0.32) | 0.12(0.08, 0.17) | 0.14(0.06, 0.31) | 0.49(0.40, 0.61) | 0.85(0.57, 1.27) | 0.67(0.32, 1.44) |
| Head of household | Male |  | 0.94(0.74, 1.20) | 0.95(0.76, 1.21) |  | 1.09(0.82, 1.45) | 0.99(0.75, 1.30) |
| Disability in household | Yes |  | 1.37(1.01, 1.84)* | 1.23(0.91, 1.66)* |  | 1.93(1.36, 2.73)* | 1.90(1.35, 2.68)* |
| Family type | Married/cohabit/engaged |  | 0.91(0.66, 1.26) | 0.86(0.63, 1.17) |  | 1.32(0.90, 1.92) | 1.24(0.86, 1.78) |
|  | Divorced/separated/widowed |  | 1.17(0.92, 1.49) | 1.09(0.86, 1.37) |  | 1.14(0.84, 1.53) | 1.16(0.86, 1.55) |
| Income activity engagement | Yes |  | 1.24(0.99, 1.57) | 1.17(0.95, 1.47) |  | 1.26(0.96, 1.67) | 1.30(1.00, 1.70)* |
| Food security | Food insecure |  | 1.43(1.13, 1.83)* | 1.36(1.07, 1.72)* |  | 1.06(0.79, 1.41) | 0.99(0.75, 1.31) |
| Community residence | Dwazark |  | 3.03(2.29, 4.02)* | 3.32(1.90, 5.83)* |  | 0.24(0.17, 0.34)* | 0.21(0.12, 0.38)* |
|  | Moyiba |  | 1.17(0.88, 1.54) | 1.68(0.96, 2.92) |  | 0.21(0.15, 0.29)* | 0.22(0.13, 0.39)* |
| Household size |  |  |  | 1.05(1.02, 1.08)* |  |  | 1.00(0.97, 1.03) |
| Length of residence | 1-5 year |  |  | 1.09(0.81, 1.48) |  |  | 1.09(0.82, 1.45) |
|  | 6-10 year |  |  | 1.16(0.85, 1.60) |  |  | 1.06(0.78, 1.43) |
|  | More than 10 years |  |  | 1.35(1.00, 1.83)* |  |  | 1.15(0.86, 1.53) |
| Household tenure | landlord |  |  | 0.81(0.67, 0.97) |  |  | 1.13(0.88, 1.44) |
|  | Free living |  |  | 0.84(0.65, 1.08) |  |  | 1.14(0.96, 1.36) |
|  | Caretaker/lease/  temporary stay/others |  |  | 0.76(0.47, 1.20) |  |  | 1.03(0.68, 1.54) |
| Piped dwelling | Yes |  |  | 0.86(0.55, 1.32) |  |  | 0.74(0.49, 1.09) |
| Piped neighbor | Yes |  |  | 2.33(1.74, 3.09)* |  |  | 1.03(0.78, 1.35) |
| Public tap/standpipe | Yes |  |  | 0.98(0.79, 1.21) |  |  | 0.82(0.67, 1.01) |
| Compound well | Yes |  |  | 0.97(0.78, 1.21) |  |  | 1.06(0.84, 1.33) |
| Spring water | Yes |  |  | 1.02(0.81, 1.28) |  |  | 1.06(0.86, 1.29) |
| Rainwater | Yes |  |  | 1.02(0.85, 1.22) |  |  | 0.66(0.55, 0.78)* |
| Bowser water | Yes |  |  | 0.88(0.53, 1.43) |  |  | 0.42(0.22, 0.75)* |
| Kiosk water | Yes |  |  | 0.95(0.69, 1.30) |  |  | 1.53(1.15, 2.03)* |
| Bottled water | Yes |  |  | 0.46(0.21, 0.93) |  |  | 1.90(1.10, 3.28)* |
| Sachet water | Yes |  |  | 0.73(0.62, 0.85) |  |  | 1.17(1.01, 1.37)* |
| Surface water | Yes |  |  | 0.74(0.57, 0.96) |  |  | 0.64(0.49, 0.84) |
| Neighbor’s well | Yes |  |  | 1.40(1.13, 1.72)* |  |  | 1.14(0.93, 1.39)* |
| Other water sources | Yes |  |  | 0.52(0.25, 1.00) |  |  | 0.82(0.49, 1.35) |
| Water distance | 30 minutes-1 hours |  |  | 0.81(0.67, 0.98) |  |  | 0.94(0.78, 1.14) |
|  | 1- 2 hours |  |  | 0.68(0.52, 0.88) |  |  | 1.33(1.04, 1.70)* |
|  | Over 2 hours |  |  | 0.56(0.45, 0.71) |  |  | 1.36(1.09, 1.70)* |
|  | Don’t know |  |  | 1.52(0.98, 2.34) |  |  |  |
| Water shortage | No |  |  | 0.83(0.70, 0.98)* |  |  | 0.80(0.68, 0.93)* |
|  | Don’t know |  |  | 0.86(0.23, 2.94) |  |  | 0.00(0.00, 0.00) |
| Flush | Yes |  |  | 1.32(0.95, 1.83) |  |  | 1.58(1.19, 2.10)* |
| Latrine | Yes |  |  | 0.62(0.52, 0.75) |  |  | 0.86(0.72, 1.02) |
| Bucket | Yes |  |  | 1.23(0.83, 1.82) |  |  | 1.37(0.97, 1.94) |
| Hanging | Yes |  |  | 1.12(0.80, 1.57) |  |  | 1.18(0.87, 1.60) |
| Open defecation | Yes |  |  | 1.25(0.72, 2.15) |  |  | 2.50(1.52, 4.20)* |
| Other (toilet types) | Yes |  |  | 0.97(0.36, 2.41) |  |  | 0.82(0.34, 1.89) |
| Shared toilet | No |  |  | 0.68(0.57, 0.83) |  |  | 1.00(0.84, 1.19) |
|  | Not applicable |  |  | 0.38(0.16, 0.86) |  |  |  |
| Around house | Yes |  |  | 1.03(0.83, 1.28) |  |  | 1.03(0.82, 1.29) |
| Dumping site | Yes |  |  | 1.25(0.93, 1.67) |  |  | 1.28(0.95, 1.71) |
| Drainage | Yes |  |  | 1.30(1.07, 1.58)* |  |  | 0.90(0.74, 1.08) |
| Solid waste collectors | Yes |  |  | 0.89(0.59, 1.37) |  |  | 0.67(0.44, 1.01) |
| Waste sea | Yes |  |  | 0.95(0.57, 1.56) |  |  | 1.10(0.67, 1.79) |
| Other (waste disposal) | Yes |  |  | 1.58(1.10, 2.27)* |  |  | 1.69(1.15, 2.45)* |
| Waste payment | No |  |  | 1.02(0.69, 1.50) |  |  | 1.43(0.98, 2.09) |
| Environmental disaster | Yes |  |  | 0.73(0.56, 0.95) |  |  | 0.84(0.65, 1.10) |
| business | Yes |  |  | 1.08(0.91, 1.28) |  |  | 1.28(1.08, 1.51)* |
| fishing | Yes |  |  | 1.27(0.58, 2.63) |  |  | 1.96(1.00, 3.80) |
| Government salaried | Yes |  |  | 0.83(0.61, 1.11) |  |  | 1.30(0.97, 1.74) |
| Private salaried | Yes |  |  | 1.31(1.05, 1.62)* |  |  | 1.32(1.06, 1.64)* |
| Daily wage | Yes |  |  | 0.77(0.57, 1.02) |  |  | 0.97(0.73, 1.28) |
| Stone mine | Yes |  |  | 0.75(0.46, 1.21) |  |  | 0.56(0.35, 0.89)* |
| Other (Income sources) | Yes |  |  | 1.02(0.75, 1.38) |  |  | 1.57(1.19, 2.07)* |
| Study sample | | 4,821 | 4,821 | 4,821 |  | 4,616 | 4616 |
| Strata number | | 122 | 122 | 122 |  | 122 | 122 |
| Strata variance | | 0.39 | 0.07 | 0.04 | 0.76 | 0.19 | 0.15 |
| VPC | | 10.64% | 1.95% | 1.14% | 18.74% | 5.56% | 4.38% |
| PCV | | - | 83.28% | 90.30% | - | 74.48% | 80.15% |

Model 1: Null model; Model 2: main effects model; Model 3: main effects and explanatory variables model; OR (95%CI): Odds Ratio (95% Confidence Interval); *: significant at 5% level; VPC: variance partitioning coefficient; PCV: proportional change in variance; reference categories excluded; “-“: explanatory variables excluded based on univariate analysis.

**Health utilisation (HU) within and outside the informal settlements**

The findings for HU within and outside settlements are presented in Supplementary Tables 8a and 9a, and Figure 1a, showing a pattern similar to that of HU within and outside settlements separately (Table 1). The VPC decreased from 11.4% in Model 1 to 3.9% in Model 2 and 2.3% in Model 3. The PCV was 68.1% for Model 2 compared to Model 1 and 82.0% for Model 3 compared to Model 3, indicating that 20.0% of the total variance between strata remained due to intersectional effects after accounting for main effects and explanatory variables.

Table 9a most intersectional effects for HU within and outside the settlements were explained by income-generating activities, food security and community of residence Significant variables associated with HU included length of residence, household size, sources of income (business, government salaried, daily wage, stone mining), tenure, water sources (piped neighbour, public tap, rain, bowser, kiosk, bottled, sachet, surface, and neighbour’s well), toilet types (flush, bucket, hanging, flying, open defecation, shared toilet), waste disposal (around house, dumping site, solid collectors), waste payment and environmental disaster. The direction of associations for each variable was consistent with those observed when considering HU within and outside settlements separately.

Moreover, the pattern for strata residuals in Figure 1a was consistent with those for HU within and outside settlements separately. The lowest HU within and outside the settlements was observed among households headed by a single male from Moyiba, without income-generating activities, who were food insecure and had a disabled person. They were followed by households with a female, divorced/separated/widowed head from Moyiba, also without income generating activities, who were food insecure and without disabled persons from Moyiba. Additionally, households headed by single disabled males from Moyiba without income-generating activities and food insecure and those with female-married/cohabiting/engaged heads also from Moyiba and engaged in income generating activities and with a disabled persons had a low HU.

Households headed by single female- from Cockle Bay, engaged in income generating activities, food secure and without disabled persons from had the highest HU within and outside settlements. They were followed by those with male, divorced/separated/widowed heads from Moyiba, also engaged in income-generating activity, food secure, and without disabled persons from Moyiba. Additionally, households headed by married/cohabiting/engaged females in Dwazark, as well as those with male- divorced/separated/widowed heads in Cockle Bay, all engaged in income generating activities, food secure, and without disabled persons, had a high HU within and outside the settlements.

Table 8a: Household characteristics by healthcare utilisation (HU) within and outside settlements community among households in Cockle Bay, Dwazark, Moyiba informal settlements.

| Variable name | | Category | HU within and outside settlements: Frequency (%) | | |
| --- | --- | --- | --- | --- | --- |
|  |  |  | No | Yes | Total |
| **Social position factors** | | | | | |
| Head of household | | Female | 630(47.4) | 700(52.6) | 1330(27.5) |
|  |  | Male | 1669(47.6) | 1839(52.4) | 3508(72.5) |
| Disability in household | | No | 2185(48.4) | 2334(51.7) | 4519(93.4) |
|  |  | Yes | 114(35.7) | 205(64.3) | 319(6.6) |
| Family type | | Single | 638(52.2) | 585(47.8) | 1223(25.3) |
|  |  | Married/cohabit/engaged | 4121(46.5) | 1636(53.5) | 3057(63.2) |
|  |  | Divorced/separated/widowed | 240(43.0) | 318(57.0) | 558(11.5) |
| Income activity engagement | | No | 611(51.3) | 579(48.66) | 1190(24.6) |
|  |  | Yes | 1688(46.3) | 1960(53.7) | 3648(75.4) |
| Food security | | Food secure | 1357(56.5) | 1047(43.6) | 2404(49.7) |
|  |  | Food insecure | 942(38.7) | 1492(61.3) | 2434(50.3) |
| Community type | | Cockle Bay | 383(34.4) | 731(65.6) | 1114(23.0) |
|  |  | Dwazark | 558(10.1) | 832(59.9) | 1390(28.7) |
|  |  | Moyiba | 1358(58.2) | 976(41.8) | 2334(48.2) |
| **Predisposing factors** | | | | | |
| Length of residence | | 0-1 years | 183(50.8) | 177(49.2) | 360(7.4) |
|  |  | 1-5 years | 747(51.8) | 694(48.2) | 1441(29.8) |
|  |  | 6-10 years | 479(49.5) | 488(50.5) | 967(20.0) |
|  |  | More than 10 years | 890(43.0) | 1180(57.0) | 2070(42.8) |
| Household tenure | | Tenant | 1288(46.2) | 1501(53.8) | 2789(57.6) |
|  |  | landlord | 703(49.0) | 733(51.0) | 1436(29.7) |
|  |  | Free living | 242(50.2) | 240(49.8) | 482(10.0) |
|  |  | Caretaker/lease/temporary stay/others | 66(50.4) | 65(49.6) | 131(2.7) |
| Water Sources | Piped dwelling | No | 2221(47.5) | 2458(52.5) | 4679(96.7) |
|  |  | Yes | 78(49.1) | 81(50.9) | 159(3.3) |
|  | Piped neighbor | No | 2204(49.1) | 2285(50.9) | 4489(92.8) |
|  |  | Yes | 95(27.2) | 254(72.8) | 349(7.2) |
|  | piped compound | No | 2246(47.9) | 2448(52.2) | 4694(97.0) |
|  |  | Yes | 53(36.8) | 91(63.2) | 144(2.9) |
|  | Public tap/standpipe | No | 1767(46.9) | 1998(53.1) | 3765(77.8) |
|  |  | Yes | 532(49.6) | 541(50.4) | 1073(22.2) |
|  | Compound well | No | 1982(48.2) | 2127(51.8) | 4109(84.9) |
|  |  | Yes | 317(43.5) | 412(56.5) | 729(15.1) |
|  | Spring water | No | 1833(47.9) | 1994(52.1) | 3827(79.1) |
|  |  | Yes | 466(46.1) | 545(53.9) | 1011(20.9) |
|  | Rainwater | No | 1382(44.1) | 1753(55.9) | 3135(64.8) |
|  |  | Yes | 917(53.85) | 786(46.15) | 1703(35.2) |
|  | Bowser water | No | 2252(47.4) | 2497(52.6) | 4749(98.2) |
|  |  | Yes | 47(52.8) | 42(47.2) | 89(1.8) |
|  | Kiosk water | No | 2157(17.5) | 2386(52.5) | 4543(93.9) |
|  |  | Yes | 142(48.1) | 153(51.9) | 295(6.1) |
|  | Bottled water | No | 2270(47.6) | 2502(52.4) | 4772(98.6) |
|  |  | Yes | 29(43.9) | 37(59.1) | 66(1.4) |
|  | Sachet water | No | 1005(49.7) | 1018(50.3) | 2023(41.8) |
|  |  | Yes | 1294(46.0) | 1521(54.0) | 2815(58.2) |
|  | Surface water | No | 1938(45.1) | 2361(54.9) | 4299(88.9) |
|  |  | Yes | 361(67.0) | 178(33.0) | 539(11.1) |
|  | Neighbor’s well | No | 2026(49.5) | 2067(50.5) | 4093(84.6) |
|  |  | Yes | 273(36.6) | 472(63.4) | 745(15.4) |
|  | Other water sources | No | 2269(47.6) | 2494(52.4) | 4763(98.5) |
|  |  | Yes | 30(40.0) | 45(60.0) | 75(1.6) |
| Water distance | | Less 30 minutes | 651(42.4) | 885(57.6) | 1536(31.7) |
|  |  | 30 minutes-1 hours | 750(51.1) | 717(48.9) | 1467(30.3) |
|  |  | 1-2 hours | 320(48.9) | 335(51.2) | 655(13.5) |
|  |  | Over 2 hours | 535(50.3) | 529(49.7) | 1064(22.0) |
|  |  | Don’t know | 43(37.1) | 73(62.9) | 116(2.4) |
| Water shortage | | Yes | 1350(45.9) | 1593(54.1) | 2943(60.8) |
|  |  | No | 942(50.0) | 943(50.0) | 1885(39.0) |
|  |  | Don’t know | 7(70.0) | 3(30.0) | 10(0.2) |
| Types of toilets | Flush | No | 1860(50.4) | 1828(49.6) | 3688(76.2) |
|  |  | Yes | 439(38.2) | 711(61.8) | 1150(23.8) |
|  | Latrine | No | 637(40.0) | 956(60.0) | 1593(32.9) |
|  |  | Yes | 1662(51.2) | 1583(48.8) | 3245(67.1) |
|  | Bucket | No | 1426(43.3) | 1868(56.7) | 3294(68.1) |
|  |  | Yes | 873(56.5) | 671(43.5) | 1544(31.9) |
|  | Hanging | No | 2190(48.2) | 2356(51.8) | 4546(93.9) |
|  |  | Yes | 109(37.3) | 183(62.7) | 292(6.0) |
|  | Flying | No | 2290(47.6) | 2526(52.4) | 4816(99.6) |
|  |  | Yes | 9(10.9) | 13(59.1) | 22(0.4) |
|  | Open defecation | No | 2276(48.1) | 2456(51.9) | 4732(97.8) |
|  |  | Yes | 23(21.7) | 83(78.3) | 106(2.2) |
|  | Other | No | 2286(47.6) | 2522(52.5) | 4808(99.4) |
|  |  | Yes | 13(43.3) | 17(56.7) | 30(0.62) |
| Shared toilet | | Yes | 1618(47.4) | 1795(52.6) | 3413(70.6) |
|  |  | No | 656(47.7) | 719(52.3) | 1375(28.4) |
|  |  | Not applicable | 25(50.0) | 25(50.0) | 50(1.0) |
| Toilet access | | Yes | 2299(47.5) | 2539(52.5) | 4104(84.8) |
|  |  | No | 326(46.0) | 382(54.0) | 708(14.6) |
|  |  | Don’t know | 17(65.4) | 9(34.6) | 26(0.5) |
| Waste disposal areas | Community | Yes | 74(41.8) | 103(58.2) | 177(3.7) |
|  |  | No | 2207(47.6) | 2427(52.4) | 4634(95.8) |
|  |  | Don’t know | 18(66.7) | 9(33.3) | 27(0.6) |
|  | Around house | No | 990(42.7) | 1331(57.4) | 2321(47.9) |
|  |  | Yes | 1309(52.0) | 1208(48.0) | 2517(52.0) |
|  | Dumping site | No | 2132(47.4) | 2370(52.6) | 4502(93.1) |
|  |  | Yes | 167(49.7) | 169(50.3) | 336(6.9) |
|  | Drainage | No | 1752(46.6) | 2011(53.4) | 3763(77.8) |
|  |  | Yes | 547(50.9) | 528(49.1) | 1075(22.2) |
|  | Solid waste collectors | No | 1817(46.1) | 2127(53.9) | 3944(81.5) |
|  |  | Yes | 482(53.9) | 412(46.1) | 894(18.5) |
|  | Waste sea | No | 1903(51.3) | 1807(48.7) | 3710(76.7) |
|  |  | Yes | 396(35.1) | 732(64.9) | 1128(23.3) |
|  | Waste others | No | 2255(48.3) | 2413(51.7) | 4668(96.5) |
|  |  | Yes | 44(25.9) | 126(74.1) | 170(3.5) |
| Waste payment | | No | 1781(46.5) | 2053(53.6) | 3834(79.3) |
|  |  | Yes | 518(51.6) | 486(48.4) | 1004(20.7) |
| Environmental disaster | | No | 130(36.5) | 226(63.5) | 356(7.4) |
|  |  | Yes | 2169(48.4) | 2313(51.6) | 4482(92.6) |
| **Enabling factors** | | | | | |
| Sources of household income | business | No | 1445(46.8) | 1641(53.2) | 3086(63.8) |
|  |  | Yes | 854(48.7) | 898(51.3) | 1752(36.2) |
|  | fishing | No | 2285(47.6) | 2513(52.4) | 4798(99.2) |
|  |  | Yes | 14(35.0) | 26(65.0) | 40(0.8) |
|  | Government salaried | No | 2151(47.7) | 2358(52.3) | 4509(93.2) |
|  |  | Yes | 148(45.0) | 181(55.0) | 329(6.8) |
|  | Private salaried | No | 2061(48.7) | 2168(21.3) | 4229(87.4) |
|  |  | Yes | 238(39.1) | 371(60.9) | 609(12.6) |
|  | Informal salaried | No | 2272(47.5) | 2511(52.5) | 4783(98.8) |
|  |  | Yes | 27(49.1) | 28(50.9) | 55(1.1) |
|  | Daily wage | No | 2098(47.0) | 2365(53.0) | 4463(92.3) |
|  |  | Yes | 201(53.6) | 174(46.4) | 375(7.6) |
|  | Bike ride | No | 2121(47.2) | 2376(52.8) | 4497(92.9) |
|  |  | Yes | 178(52.2) | 163(47.8) | 341(7.1) |
|  | Stone mine | No | 2205(47.0) | 2484(53.0) | 4689(96.9) |
|  |  | Yes | 94(63.1) | 55(33.9) | 149(3.1) |
|  | Unemployed | No | 2295(47.5) | 2535(52.5) | 4830(99.8) |
|  |  | Yes | 4(50.0) | 4(50.0) | 8(0.2) |
|  | Others | No | 2185(48.3) | 2335(51.7) | 4520(93.4) |
|  |  | Yes | 114(35.9) | 204(34.2) | 318(6.6) |
| Total | |  | 2299(47.5) | 2539(52.5) | 4838(100.0) |

Table 9a: Coefficient estimates from multilevel models of health utilisation (HU) within and outside settlements among households of Cockle Bay, Dwazark and Moyiba informal settlements.

|  |  | HU within and outside the settlement | | |
| --- | --- | --- | --- | --- |
|  |  | Model 1 | Model 2 | Model 3 |
|  | Category: reference excluded | OR (95%CI) | OR (95%CI) | OR (95%CI) |
|  | Intercept | 1.20(1.02, 1.42) | 1.10(0.77, 1.57) | 1.09(0.56, 2.13) |
| Head of household | Male |  | 0.99(0.77, 1.26) | 0.92(0.73, 1.15) |
| Disability in household | Yes |  | 1.80(1.31, 2.49) | 1.66(1.22, 2.28)* |
| Family type | Married/cohabit/engaged |  | 1.43(1.03, 1.99)* | 1.33(0.98, 1.81) |
|  | Divorced/separated/widowed |  | 1.22(0.93, 1.58)* | 1.20(0.95, 1.52) |
| Income activity engagement | Yes |  | 1.35(1.09, 1.70) | 1.33(1.08, 1.66)* |
| Food security | Food insecure |  | 1.25(0.97, 1.59) | 11.16(0.91, 1.45)* |
| Community residence | Dwazark |  | 0.74(0.55, 1.01) | 0.83(0.51, 1.37) |
|  | Moyiba |  | 0.34(0.25, 0.45)* | 0.48(0.29, 0.78)* |
| Household size |  |  |  | 1.03(1.01, 1.06)* |
| Length of residence | 1-5 year |  |  | 0.99(0.77, 1.27) |
|  | 6-10 year |  |  | 1.09(0.84, 1.42) |
|  | More than 10 years |  |  | 1.36(1.06, 1.75)* |
| Household tenure | landlord |  |  | 0.83(0.71, 0.96)* |
|  | Free living |  |  | 1.00(0.80, 1.24) |
|  | Caretaker/lease/  temporary stay/others |  |  | 0.73(0.49, 1.07) |
| Piped dwelling | Yes |  |  | 0.85(0.59, 1.22) |
| Piped neighbor | Yes |  |  | 1.58(1.20, 2.08)* |
| Public tap/standpipe | Yes |  |  | 0.91(0.76, 1.09) |
| Compound well | Yes |  |  | 0.87(0.71, 1.07) |
| Spring water | Yes |  |  | 1.03(0.85, 1.25) |
| Rainwater | Yes |  |  | 0.68(0.59, 0.80)* |
| Bowser water | Yes |  |  | 0.55(0.35, 0.86)* |
| Kiosk water | Yes |  |  | 1.06(0.82, 1.37) |
| Bottled water | Yes |  |  | 1.25(0.74, 2.10) |
| Sachet water | Yes |  |  | 1.00(0.88, 1.15) |
| Surface water | Yes |  |  | 0.53(0.42, 0.66) |
| Neighbor’s well | Yes |  |  | 1.29(1.07, 1.55)* |
| Other water sources | Yes |  |  | 0.81(0.49, 1.34) |
| Water distance | 30 minutes-1 hours |  |  | 0.91(0.77, 1.07) |
|  | 1- 2 hours |  |  | 1.04(0.84, 1.30) |
|  | Over 2 hours |  |  | 1.04(0.85, 1.27) |
|  | Don’t know |  |  | 1.40(0.91, 2.17) |
| Water shortage | No |  |  | 0.76(0.67, 0.88)* |
|  | Don’t know |  |  | 0.67(0.21, 2.00) |
| Flush | Yes |  |  | 1.62(1.24, 2.13)* |
| Latrine | Yes |  |  | 0.78(0.67, 0.91)* |
| Bucket | Yes |  |  | 1.45(1.04, 2.03)* |
| Hanging | Yes |  |  | 1.24(0.94, 1.65) |
| Open defecation | Yes |  |  | 2.49(1.50, 4.20*) |
| Other (toilet types) | Yes |  |  | 1.38(0.65, 2.93)* |
| Shared toilet | No |  |  | 0.88(0.75, 1.03) |
|  | Not applicable |  |  | 0.71(0.39, 1.27) |
| Around house | Yes |  |  | 1.16(0.95, 1.41) |
| Dumping site | Yes |  |  | 1.24(0.96, 1.60) |
| Drainage | Yes |  |  | 1.01(0.86, 1.20) |
| Solid waste collectors | Yes |  |  | 0.58(0.39, 0.86)* |
| Waste sea | Yes |  |  | 1.25(0.82, 1.92) |
| Other (waste disposal) | Yes |  |  | 2.34(1.60, 3.46)* |
| Waste payment | Yes |  |  | 1.36(0.95, 1.96)* |
| Environmental disaster | Yes |  |  | 0.61(0.48, 0.78)* |
| business | Yes |  |  | 1.21(1.04, 1.39)* |
| fishing | Yes |  |  | 1.65(0.86, 3.22) |
| Government salaried | Yes |  |  | 1.14(0.88, 1.48) |
| Private salaried | Yes |  |  | 1.42(1.16, 1.74)* |
| Daily wage | Yes |  |  | 0.95(0.75, 1.21) |
| Stone mine | Yes |  |  | 0.71(0.49, 1.03) |
| Other (Income sources) | Yes |  |  | 1.55(1.18, 2.03)* |
| Study sample | | 4,838 | 4,838 | 4,838 |
| Strata number | | 122 | 122 | 122 |
| Strata variance | | 0.42 | 0.06 | 0.03 |
| VPC | | 11.38% | 3.93% | 2.26% |
| PCV | | - | 68.12% | 81.97% |

Model 1: Null model; Model 2: main effects model; Model 3: main effects and explanatory variables model; OR (95%CI): Odds Ratio (95% Confidence Interval); *: significant at 5% level of significance; VPC: variance partitioning coefficient; PCV: proportional change in variance


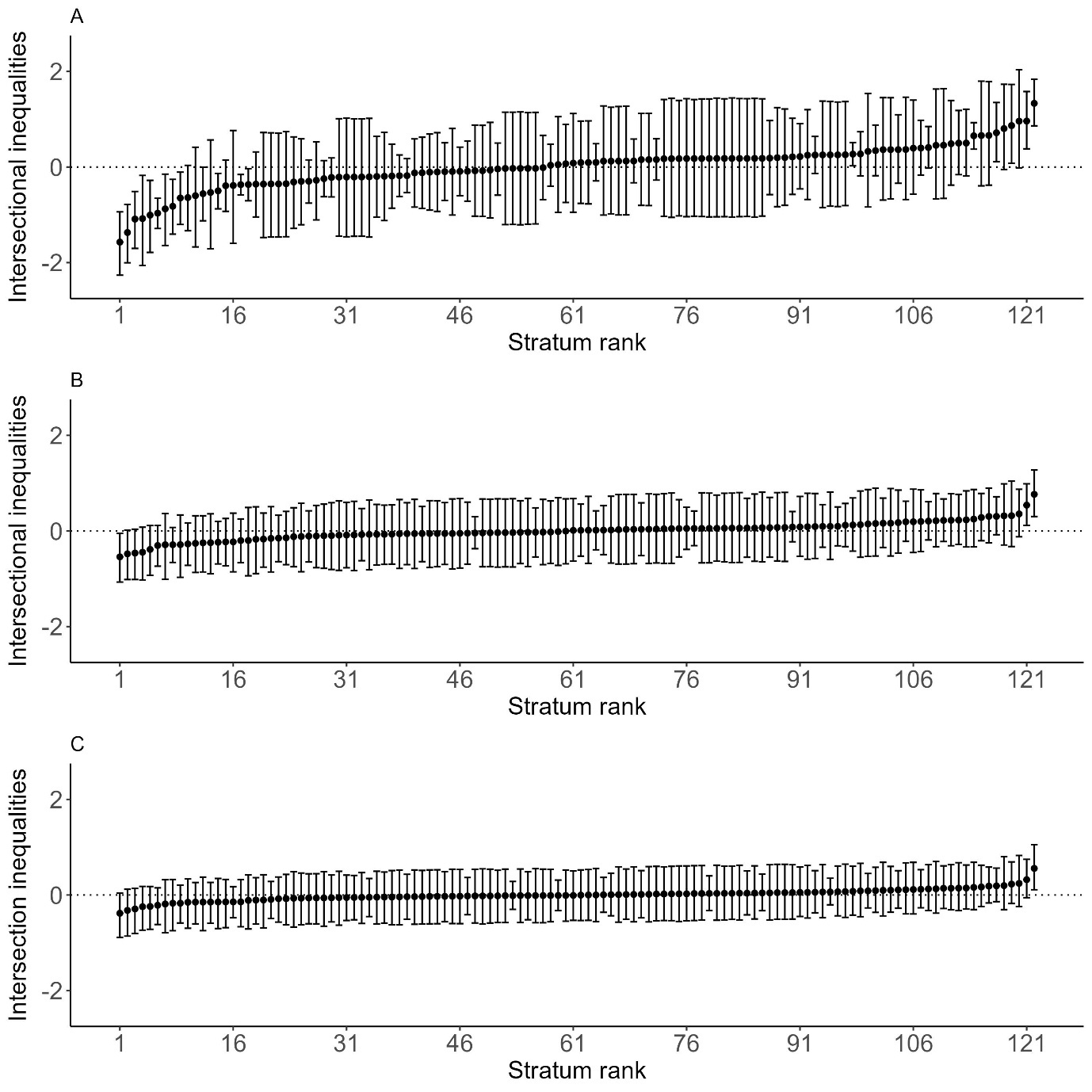


Figure 3a: Estimated intersectional effects residual estimates and their corresponding 95% credible intervals (CI) for each stratum for HU within and outside the settlements ranked from lowest to highest: Model 1 (Panel A), Model 2 (Panel B) and Model 3 (Panel C).

Table 10a: Diagnostic measures assessing the quality of posterior estimates for HU within and outside settlements in Model 1: Rhat, Bulk effective sample size (ESS) and tail ESS.

|  |  | HU within and outside the settlement | | |
| --- | --- | --- | --- | --- |
|  |  | Rhat | Bulk ESS | Tail ESS |
|  | Intercept | 1.00 | 8500.43 | 15458.81 |
| Random intercept | | 1.00 | 10520.10 | 20068.55 |

Model 1: Null model

Table11a: Diagnostic measures assessing the quality of posterior estimates for HU within and outside settlements in Model 2: Rhat, Bulk effective sample size (ESS) and tail ESS.

|  |  | HU within the settlement | | | |
| --- | --- | --- | --- | --- | --- |
|  |  | Rhat | Bulk ESS | Tail ESS |  |
|  | Intercept | 1.00 | 9353.77 | 15754.48 |  |
| Head of household | Male | 1.00 | 15754.21 | 22684.20 |  |
| Disability in household | Yes | 1.00 | 20154.74 | 25059.79 |  |
| Family type | Married/cohabit/engaged | 1.00 | 18130.68 | 23838.74 |  |
|  | Divorced/separated/widowed | 1.00 | 14365.46 | 20708.71 |  |
| Income activity engagement | Yes | 1.00 | 15160.05 | 22155.26 |  |
| Food security | Food insecure | 1.00 | 16281.5 | 22738.33 |  |
| Community residence | Dwazark | 1.00 | 14595.91 | 20022.34 |  |
|  | Moyiba | 1.00 | 13617.34 | 21096.37 |  |
| Random intercept | | 1.00 | 1.00 | 20614.12 |  |

Model 2: main effects model variables

Table 12a: Diagnostic measures assessing the quality of posterior estimates for HU within and outside informal settlements in model 3: Rhat, Bulk effective sample size (ESS) and tail ESS.

|  |  | HU within and outside the settlement | | |
| --- | --- | --- | --- | --- |
|  |  | Rhat | Bulk ESS | Tail ESS |
|  | Intercept | 1.00 | 26918.47 | 25347.15 |
| Head of household | Male | 1.00 | 43619.05 | 27293.04 |
| Disability in household | Yes | 1.00 | 48577.4 | 29648.03 |
| Family type | Married/cohabit/engaged | 1.00 | 47613.00 | 28086.49 |
|  | Divorced/separated/widowed | 1.00 | 33689.78 | 27653.14 |
| Income activity engagement | Yes | 1.00 | 37991.58 | 29448.73 |
| Food security | Food insecure | 1.00 | 38653.28 | 28585.4 |
| Community residence | Dwazark | 1.00 | 34547.82 | 27207.17 |
|  | Moyiba | 1.00 | 36089.84 | 28172.37 |
| Household size |  | 1.00 | 73398.95 | 28657.66 |
| Length of residence | 1-5 year | 1.00 | 36842.73 | 29478.39 |
|  | 6-10 year | 1.00 | 34689.77 | 28287.74 |
|  | More than 10 years | 1.00 | 34732.86 | 28211.89 |
| Household tenure | landlord | 1.00 | 67360.63 | 28962.22 |
|  | Free living | 1.00 | 74822.37 | 26256.49 |
|  | Caretaker/lease/  temporary stay/others | 1.00 | 86029.58 | 26517.12 |
| Piped dwelling | Yes | 1.00 | 85466.98 | 25949.44 |
| Piped neighbor | Yes | 1.00 | 86366.58 | 27262.94 |
| Public tap/standpipe | Yes | 1.00 | 58543.46 | 30633.38 |
| Compound well | Yes | 1.00 | 59041.45 | 30482.23 |
| Spring water | Yes | 1.00 | 57536.02 | 30594.25 |
| Rainwater | Yes | 1.00 | 53948.27 | 30747.06 |
| Bowser water | Yes | 1.00 | 84129.87 | 25350.74 |
| Kiosk water | Yes | 1.00 | 79003.39 | 25505.53 |
| Bottled water | Yes | 1.00 | 94166.4 | 24725.73 |
| Sachet water | Yes | 1.00 | 80601.79 | 27544.04 |
| Surface water | Yes | 1.00 | 72721.44 | 28176.56 |
| Neighbor’s well | Yes | 1.00 | 67955.71 | 28284.64 |
| Other water sources | Yes | 1.00 | 82486.2 | 27103.92 |
| Water distance | 30 minutes-1 hours | 1.00 | 51774.46 | 30486.98 |
|  | 1- 2 hours | 1.00 | 48518.56 | 30573.48 |
|  | Over 2 hours | 1.00 | 46992.57 | 31307.83 |
|  | Don’t know | 1.00 | 64782.39 | 28384.67 |
| Water shortage | No | 1.00 | 75524.19 | 27192.51 |
|  | Don’t know | 1.00 | 94464.53 | 25982.4 |
| Flush | Yes | 1.00 | 40911.29 | 30484.31 |
| Latrine | Yes | 1.00 | 61322.32 | 29112.61 |
| Bucket | Yes | 1.00 | 49584.07 | 31538.35 |
| Hanging | Yes | 1.00 | 39604.98 | 30215.9 |
| Open defecation | Yes | 1.00 | 69135.29 | 26005.98 |
| Other (toilet types) | Yes | 1.00 | 70703.69 | 26451.6 |
| Shared toilet | No | 1.00 | 76662.11 | 28516.01 |
|  | Not applicable | 1.00 | 86540.5 | 25110.45 |
| Around house | Yes | 1.00 | 59897.34 | 29025.44 |
| Dumping site | Yes | 1.00 | 66033.93 | 30777.93 |
| Drainage | Yes | 1.00 | 67089.75 | 28096.46 |
| Solid waste collectors | Yes | 1.00 | 52673.71 | 28413.08 |
| Waste sea | Yes | 1.00 | 40374.55 | 29559.57 |
| Other (waste disposal) | Yes | 1.00 | 76839.08 | 28460.99 |
| Waste payment | No | 1.00 | 56183.07 | 28771.57 |
| Environmental disaster | Yes | 1.00 | 86737.3 | 26362.34 |
| business | Yes | 1.00 | 64135.88 | 30438.12 |
| fishing | Yes | 1.00 | 91981.2 | 26169.9 |
| Government salaried | Yes | 1.00 | 76716.8 | 28625.19 |
| Private salaried | Yes | 1.00 | 70764.45 | 30381.31 |
| Daily wage | Yes | 1.00 | 74062.91 | 26246.43 |
| Stone mine | Yes | 1.00 | 82676.25 | 25602.21 |
| Other (Income sources) | Yes | 1.00 | 1.00 | 27666.92 |
| Radom intercept | | 1.00 | 1.00 | 1.00 |

Figure 2a: Trace plot showing sampled values of the standard deviation and posterior density plot showing the distribution of estimated values for the random intercept (intersectional strata) in health utilisation within and outside informal settlements (Model 3)
